# Identification and characterization of plastid-type proteins from sequence-attributed features using machine learning

**DOI:** 10.1186/1471-2105-14-S14-S7

**Published:** 2013-10-09

**Authors:** Rakesh Kaundal, Sitanshu S Sahu, Ruchi Verma, Tyler Weirick

**Affiliations:** 1 National Institute for Microbial Forensics & Food and Agricultural Biosecurity (NIMFFAB), Department of Biochemistry & Molecular Biology, Oklahoma State University, Stillwater, OK,74078, USA; 2 Department of Biochemistry & Molecular Biology, Oklahoma State University, Stillwater, OK 74078, USA

## Abstract

**Background:**

Plastids are an important component of plant cells, being the site of manufacture and storage of chemical compounds used by the cell, and contain pigments such as those used in photosynthesis, starch synthesis/storage, cell color *etc*. They are essential organelles of the plant cell, also present in algae. Recent advances in genomic technology and sequencing efforts is generating a huge amount of DNA sequence data every day. The predicted proteome of these genomes needs annotation at a faster pace. In view of this, one such annotation need is to develop an automated system that can distinguish between plastid and non-plastid proteins accurately, and further classify plastid-types based on their functionality. We compared the amino acid compositions of plastid proteins with those of non-plastid ones and found significant differences, which were used as a basis to develop various feature-based prediction models using similarity-search and machine learning.

**Results:**

In this study, we developed separate Support Vector Machine (SVM) trained classifiers for characterizing the plastids in two steps: first distinguishing the plastid vs. non-plastid proteins, and then classifying the identified plastids into their various types based on their function (chloroplast, chromoplast, etioplast, and amyloplast). Five diverse protein features: amino acid composition, dipeptide composition, the pseudo amino acid composition, N_terminal_-Center-C_terminal _composition and the protein physicochemical properties are used to develop SVM models. Overall, the dipeptide composition-based module shows the best performance with an accuracy of 86.80% and Matthews Correlation Coefficient (MCC) of 0.74 in phase-I and 78.60% with a MCC of 0.44 in phase-II. On independent test data, this model also performs better with an overall accuracy of 76.58% and 74.97% in phase-I and phase-II, respectively. The similarity-based PSI-BLAST module shows very low performance with about 50% prediction accuracy for distinguishing plastid vs. non-plastids and only 20% in classifying various plastid-types, indicating the need and importance of machine learning algorithms.

**Conclusion:**

The current work is a first attempt to develop a methodology for classifying various plastid-type proteins. The prediction modules have also been made available as a web tool, PLpred available at http://bioinfo.okstate.edu/PLpred/ for real time identification/characterization. We believe this tool will be very useful in the functional annotation of various genomes.

## Background

**Figure 1:**
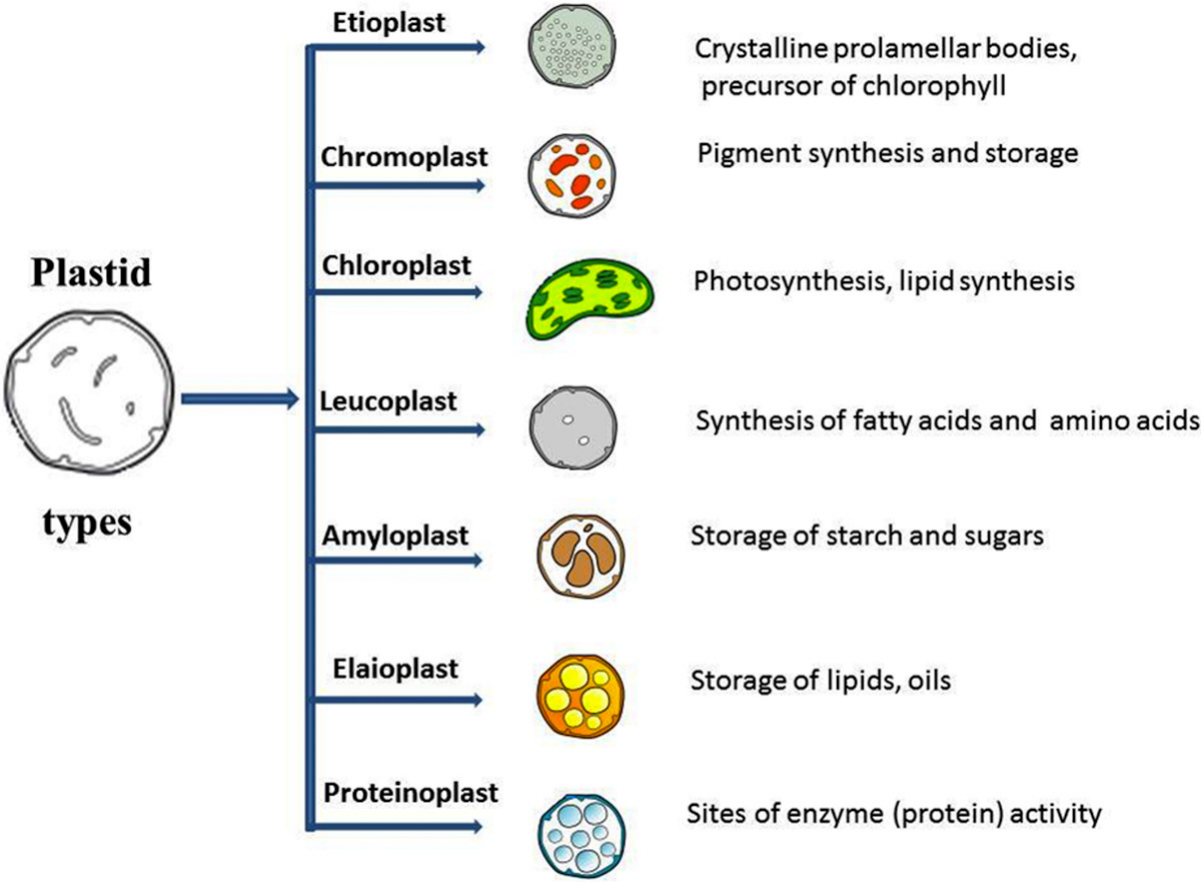
**Plastid and its various types with their respective organelle function**.

One of the major organelles in the plant cell is plastids; they perform essential biosynthetic and metabolic functions [1]. These functions include photosynthetic carbon fixation, synthesis of amino acids, fatty acids, starch and secondary metabolites such as pigments [2]. On the basis of their structure, pigment composition (color), metabolism and function, plastids are classified as 'chloroplasts' in photo-synthetically active tissues, 'chromoplasts' in fruits and petals, 'amyloplasts' in roots, 'etioplasts' in dark-grown seedlings and 'elaioplasts' that are found in the seed endosperm (Figure 1). Though plastids are of significant biological interest, current understanding of the metabolic functions and capacities of different plastid types is still limited [3]. Proteomics is a powerful approach to map the complete set of plastid proteins, and to infer plastid-type specific metabolic functions as well. Over the years, several proteomic analyses of plastids have been reported [4-11], although these come with limitations. Besides being time consuming, the experimental approaches face other constraints; for example, chloroplast proteome analysis is nearing saturation because the detection of new proteins is constrained by highly abundant photosynthetic proteins that dominate the proteome of photosynthetically active chloroplasts [12]. This has become more evident recently where the identical (or nearly identical) set of chloroplast proteins were repeatedly identified in different studies, whereas the reported detection rate of new proteins is small [13,14].

Moreover, in cases such as the ordered rearrangement of the proteome during plastid differentiation, profiling of static proteomes provides only limited information on proteome dynamics [1]. To circumvent these constraints and to increase proteome coverage, the development of highly efficient computational prediction tools is another complementary approach to provide useful global information about the plastid proteomes. Various proteomic approaches have led to the development of some databases available for plant plastids, for example, the Chloroplast Genome Database [2], plprot [13], PPDB [15]. However, there is no computational prediction system to identify and characterize various plastid types that could be used to classify '*unknown*' proteins. TargetP is currently the most widely known prediction program with a tested prediction accuracy around 68% for known plastid proteins, suggesting that a significant number of proteins cannot be identified by this type of analysis [12,16-19]. The most likely reason for this low performance is that TargetP is based on the presence of an N-terminal transit peptide region in a protein. In cases where there are alternate signals, it will fail to predict. It has been reported that plastid protein dynamics most likely also relate to different protein-targeting routes that exist in plastids [20]. This means that novel algorithms have to be developed based on whole amino acid sequence properties. Secondly, TargetP cannot predict the plastid type of a query protein e.g. whether it is a chloroplast, chromoplast, etioplast or an amyloplast protein. Previous attempts to predict plastid-types have been unsuccessful; several etioplast proteins are not predicted by TargetP for plastid localization [21].

In the current study, we have developed a prediction system for the genome-wide identification and classification of plastid proteins. This method works in two phases: first, distinction between plastid and non-plastid proteins, and second, classification of the identified plastid proteins into sub-classes (chloroplast, chromoplast, etioplast, and amyloplast). Various features of a protein sequence viz. Amino acid composition (AAC), Dipeptide composition (DIPEP), Pseudo Amino Acid Composition (PseAAC), N_terminal_-Center-C_terminal _(NCC) composition, and Physicochemical properties are explored in a Support Vector Machine (SVM) framework to develop diverse prediction models. In addition, the models have been tested on 'independent test' datasets for better confidence and reliability. An online tool, PLpred has also been developed for use by the research community. With the advances in recent genomics technology and more and more genomes being sequenced, there has been a spur in data generation lately. The predicted proteomes of these genomes thus need annotation at a much faster pace. We have developed a prediction method trained on '*known*' plastid proteins, which could be used to annotate the '*unknown*' proteins predicted from these genomic DNA sequences. We believe the current method would be a useful resource in this direction.

## Methods

### Dataset preparation

As the current method is developed in two phases, we discuss below the data collection and preparation separately. Data was collected accordingly from various online repositories.

(i). **Phase-I **(plastid vs. non-plastid): The amino acid sequences belonging to plastids were downloaded from the UniProt database (http://www.uniprot.org) by searching [keywords: plastids AND reviewed: yes], which gave 17,514 sequence hits. A similar number was collected for non-plastids by considering a combination of various classes such as nucleus, mitochondria, cytoplasm, Golgi body, cell membrane, peroxisome, vacuole, etc. However, the sequence number drastically reduced to 3535 in plastids and 3191 for non-plastids after we put a sequence identity cutoff of <30% (Table 1) on each of them using BlastClust [22]. To avoid homology bias in machine learning, a 25 or 30% sequence identity cutoff threshold is needed to guarantee that none of the proteins included in the benchmark datasets has greater than this threshold identity to any other sequences in the dataset [23-30]. This was done within class as well as across the classes. Further, about 10% of the data (316 sequences each for plastids and non-plastids) was kept aside for later independent testing of the models. Testing on independent datasets that are not used in a machine learning process has been reported to be the best benchmark to test the performance of various prediction models [29,30]. Finally, 2844 plastid and 2844 non-plastid sequences were used as positive and negative training sets, respectively for developing the models (Table 1).

**Table 1:** Number of protein sequences for plastids and non-plastid class used in phase-I (identification) training/testing

Type	Available	< 30% cutoff (within class)	< 30% cutoff (across class)	10% independent test set	Training set
Plastids	17514	3535	3160	316	2844
Non-plastids	17514	3191	3160	316	2844

**Total**	**35,028**	**6726**	**6320**	**632**	**5688**

(ii). **Phase-II **(plastid-types): A thorough search was performed in various databases such as UniProt, NCBI (http://www.ncbi.nlm.nih.gov/), plprot (http://www.plprot.ethz.ch/), PPDB (http://ppdb.tc.cornell.edu/) to search proteins of various plastid types *viz*. chloroplast, chromoplast, etioplast, amyloplast, leucoplast, elaioplast, proteinoplast *etc*. As expected, we found enough hits for 'chloroplast' as compared to other classes (Table 2). So to increase the number of sequences in other classes, we manually searched through literature related to proteomics studies in plastid types [5-10,13,21]. These sequences were carefully curated to each class and finally, a training set of four plastid types (chloroplast, chromoplast, etioplast, amyloplast) was generated to develop prediction models for plastid characterization (Table 3). These were further subjected to BlastClust analysis for <30% identity cutoff and an independent test data set was kept aside, as was done earlier in phase-I data preparation. As a result, in phase-II, 542 sequences for chloroplast, 177 for chromoplast, 220 for etioplast and 232 for amyloplast were used as a training set for classification (Table 3).

**Table 2:** Number of sequences available for plastid types in various online databases

	UniProt	NCBI	PLprot	PPDB
Chloroplast	15203	47346	690	2115
Chromoplast	75	91	143	11
Etioplast	56	21	240	0
Amyloplast	78	106	0	0
Leucoplast	2	3	0	0
Elaioplast	1	1	0	0
Proteinoplast	0	0	0	0

**Total**	**15,415**	**47,568**	**1073**	**2126**

**Table 3:** Number of protein sequences for various plastid types used in phase-II (classification) training/testing

Plastid type	Available	< 30% cutoff	10% independent test set	Training set
Chloroplast	690	602	60	542
Chromoplast	220	194	17	177
Etioplast	270	244	24	220
Amyloplast	313	255	23	232

**Total**	**1493**	**1295**	**124**	**1171**

### Feature representation methods

The following diverse features were extracted from the protein sequences for use in a machine learning framework for developing prediction models in both phases:

### Amino acid composition (AAC)

In this type of representation, each protein is defined by a 20-dimensional feature vector in Euclidean space. The protein corresponds to a point whose co-ordinates are given by the occurrence frequencies of the 20 constituent amino acids [29,31]. For a query protein *x*, let f(x_i_) represents the occurrence frequencies of its 20 constituent amino acids. Hence the composition of the amino acids (P_x_) in the query protein is given by,

(1) $$P\left(x_{i}\right)=\frac{f(x_{i})}{\sum_{i=1}^{20}f(x_{i})}\;i=1,2,3\ldots ...20$$

Hence, the protein *x *in the composition space is defined as:

$$\text{P}\left(\text{x}\right)=\left[{\text{P}}_{\text{1}}\left(\text{x}\right),\;{\text{P}}_{\text{2}}\left(\text{x}\right),\;\;\ldots \;\;,\;{\text{P}}_{\text{2}0}\left(\text{x}\right)\right]$$

### Dipeptide composition (DIPEP)

To capture the global information about the protein sequence the dipeptide composition has been used for prediction of several protein's attributes such as structure, function and location [29,30,32]. In this representation, the occurrence frequencies of each dipeptide in the sequence is computed producing a fixed pattern length of 400 (20 × 20) for the query protein. Thus, the composition of the dipeptide is given as:

(2) $$P\left(x_{i},x_{j}\right)=\frac{f(x_{i},x_{j})}{\sum_{i=1}^{20}\sum_{j=1}^{20}f(x_{i},x_{j})}\;\;\;\;\;\;\;\;i,j=1,2,3\ldots ...20$$

where *P(x_i_,x_j_)* is the fraction of each *(x_i_,x_j_)* dipeptide and *f(x_i_,x_j_)* is the frequency of occurrence of *(x_i_,x_j_)* dipeptides, and the denominator represents the total number of all possible dipeptides.

### Pseudo amino acid composition (PseAAC)

In composition based methods, protein sequence order and length information are completely lost, which in turn may affect the prediction accuracy of the model. To include all the details of its sequence order and length, Chou [33] proposed an effective way of representing known proteins as pseudo amino acid compositions (PseAAC) in his seminal study.

In this representation, the protein character sequence is coded by some of its physicochemical properties. Since the amphiphilic property (hydrophobicity and hydrophilicity) plays a very important role in protein folding, and functioning [34,35], these two indices may be used to reflect effectively the sequence order effects.

Accordingly a protein sample (P) of length 'L' is represented in PseAAC form as:

(3) $$P=\left[\begin{array}{c} \;\;p_{1}\\ \;\vdots \\ \;p_{20}\\ p_{20+1}\\ \;\vdots \\ P_{20+2\lambda } \end{array}\right]$$

where

(4) $$p_{u}=\{\begin{array}{c} \frac{f_{u}}{\sum_{i=1}^{20}f_{i}+w\sum_{j=1}^{2\lambda }\theta_{\tau }}\;\;\;\;\;\;\;\;1\leq u\leq 20)\\ \frac{w\theta_{u}}{\sum_{i=1}^{20}f_{i}+w\sum_{j=1}^{2\lambda }\theta_{\tau }}\;\;\;\;\;\;\;(20+1\leq u\leq 20+2\lambda ) \end{array}$$

and

(5) $$\theta_{\tau }=\frac{1}{L-\tau }\;\sum_{i=1}^{L-\tau }\emptyset \left(P_{i},P_{i+\tau }\right)\;\;\;\;\;\;\;\;\;\;\tau =1,2,\ldots \ldots \lambda \;and\;\lambda <L$$

$$\emptyset \left(P_{i},P_{j}\right)=H\left(P_{i}\right)\times H\left(P_{j}\right)$$

where f_i_, i = 1, 2, ..., 20 are the normalized occurrence frequencies corresponding to 20 native amino acids in the protein P, the symbol θ_τ_ represents the j-tier sequence correlation factor computed using (4) with H(P_i_) and H(P_j_) representing hydrophobic and hydrophilic values of the amino acids P_i _and P_j _respectively and the symbol 'w' represents the weight factor, which governs the degree of the sequence order effect to be incorporated. In the present study, we have judicially chosen the weight as 0.1 and as 5 for better accuracy. In essence, the first 20 values in (3) represent the classic amino acid composition, the next 2λ values reflect the amphiphilic sequence correlation along the protein chain.

### Terminal-based N-Center-C (NCC) amino acid composition

Many proteins in the cell contain important signal peptides at their N- or C-terminal region, which play as a marker for the subcellular location of the protein [30]. In this method, the amino acid composition of the N-terminal region, the C-terminal region, and the remaining center portion of protein sequence is computed separately and then concatenated together to represent a sample protein. The rationale is to provide more feature information to the SVM model based on the fact that percentage composition of a whole sequence may not give adequate weight to the compositional bias, which is known to be present in the protein terminus [29]. In this technique, a protein sample is represented as:

(6) $$P\left(x\right)=\left[AAC_{N-terminal}AAC_{Center\;region}AAC_{C-erminal}\right]$$

The AAC for each segment is computed using (1). Hence, a 60 dimensional feature vector is used to represent a protein. In an empirical study, the residue length of 25 was found to be the best compromise, both in phase-I and phase-II predictions.

### Physicochemical property-based composition

The physicochemical properties of amino acids have been successfully used to predict protein function, structure, and subcellular locations [41,53]. In this study, we grouped the amino acids of a protein into twenty physicochemical classes such as the charged residues, hydrophilic (polar) and neutral, basic polar or positively charged, acidic polar or negatively charged, aliphatic, aromatic, small, tiny, large, hydrophobic (non-polar) and aromatic, hydrophobic (non-polar) and neutral, amidic (contains amide group), cyclic, hydroxylic, sulfur-containing, h-bonding, acidic and their amide, ionizable, forms covalent cross-link (disulfide bond), and theoretical pI (isoelectric point). A detailed description of these classes is provided in Table 4. The composition of amino acids in each class is calculated as a feature to represent the protein. Thus, each protein in this method is represented by a 20 dimensional feature vector.

**Table 4:** Physicochemical properties used to represent a protein for predicting plastids and their types using SVM.

**Sr. No**.	Physicochemical property	Amino acids	# feature
1	Charged residues	D, R, E, K, H	1
2	Hydrophilic (polar) and neutral	N, Q, S, T, Y	1
3	Basic polar or Positively charged	H, K, R	1
4	Acidic polar or Negatively charged	D, E	1
5	Aliphatic	A, G, I, L, V	1
6	Aromatic	F, W, Y	1
7	Small	T, D, N	1
8	Tiny	G, A, S, P	1
9	Large	F, R, W, Y	1
10	Hydrophobic (non-polar) and aromatic	W, F	1
11	Hydrophobic (non-polar) and neutral	A, C, G, I, L, M, F, P, W, V	1
12	Amidic (*contains amide group*)	N, Q	1
13	Cyclic	P	1
14	Hydroxylic	S, T	1
15	Sulfur-containing	C, M	1
16	H-bonding	C, W, N, Q, S, T, Y, K, R, H, D, E	1
17	Acidic and their Amide	D, E, N, Q	1
18	Ionizable	D, E, H, C, Y, K, R	1
19	Forms covalent cross-link (disulfide bond)	C	1
20	Theoretical pI (isoelectric point)	-	1

### Similarity search-based PSI-BLAST module

In this study, we also performed PSI-BLAST based predictions in which a query sequence is searched based on its similarity against the non-redundant database; all of the UniProt/Swiss-Prot used as a target database. Previous studies have suggested that PSI-BLAST has the capability to detect remote homologies, and is thus preferred over the normal BLAST. It carries out an iterative search in which sequences found in one round are used to build a new score model for the next round of searching [36]. Three iterations were carried out at a best cut-off E-value of 0.001. This module was run separately for plastid and non-plastid data and the various plastid-type classes depending upon the similarity of the query protein to the proteins in the dataset. The module would return "*unknown protein type*" if no significant similarity is obtained. Accordingly, values for H (number of total hits), C (number of correct hits), P (percent of correct hits), and A (percent accuracy) are calculated to evaluate the PSI-BLAST based prediction performance.

### Support Vector Machine (SVM)

Support Vector Machine is a class of learning machines based on optimization principle from statistical learning theory, originally introduced by Vapnik and co-workers [37,38] about two decades ago. It has been well studied and extensively applied in the areas of pattern recognition, regression and classification problems in various fields of science and engineering, for example: predicting protein subcellular localization [19,29,30,32,39-42], classifying microarray data [43], predicting protein secondary structure [44,45], forecasting disease [46], predicting membrane protein type [47] and many other areas. In classification problems, the objective of SVM is to separate the training data with a maximum margin while maintaining reasonable computing efficiency. To handle the multi-class classification, a simple strategy is used by reducing the multi-classification to a series of binary classifications. The popular methods include One-Versus-Rest (OVR), One-Versus-One (OVO), and Directed Acyclic Graph Support Vector Machines (DAGSVM). In this work, we followed the OVO method for the multi-classification problem. More details of the theory of SVM have been described elsewhere [37,38].

To develop various classifiers, we have used SVM_light [48], a freely downloadable package of SVM (http://svmlight.joachims.org/). This software enables the user to define a number of parameters besides allowing a choice of built-in kernel functions, including linear, polynomial, and radial basis function (RBF). In our preliminary study, it was elucidated that the RBF kernel performed better than the linear and polynomial kernels (*data not shown*). Therefore, we used the RBF kernel in all further analysis and have presented the results accordingly.

**Training/testing schema: **In both steps, the training data was transformed into a five-fold cross-validation scheme, where the dataset is divided into five different parts. Four parts are combined to form one training set and the models developed from this set are then tested on the fifth part (called testing set). This process is repeated five times changing the training/testing set each time, and is thus called five-fold cross-validation. In addition, we have also tested the performance of our models on independent test datasets, those that have not been used in any kind of machine learning.

**Evaluation parameters**: The performance of models developed in both the phase-I (single class) and phase-II (multi class) predictions is evaluated based on the following standard parameters:

Sensitivity or coverage of positive examples: It is the percent of plastid proteins correctly predicted,

(7) $$\text{Sensitivity}\left(S_{n}\right)=\frac{TP}{TP+FN}$$

Specificity or coverage of negative examples: It is percent of non-plastids correctly predicted as non-plastid proteins,

(8) $$\text{Specificity}(Sp)=\frac{TN}{TN+FP}\;x\;100$$

Accuracy: It is the percentage of correctly predicted proteins (plastids and non-plastids proteins),

(9) $$\text{Accuracy}(Acc)=\frac{TP+TN}{TP+FN+FP+FN}x100$$

Precision: It is the percentage of positive predictions those are correct calculated as:

(10) $$\text{Precision}=\frac{TP}{TP+FP}\text{x}100$$

Rate of False Predictions (RFP): also known as False Discovery Rate (FDR), is the *expected *percent of false predictions in the set of predictions,

(11) $$\text{Rate of False Prediction}\left(\text{RFP}\right)=\frac{FP}{TP+FP}\text{x}100$$

Error Rate: gives an overall idea about the total percentage of wrong predictions calculated as:

(12) $$\text{Error Rate}\left(\text{ER}\right)=\frac{FP+FN}{TP+FN+FP+TN}\;\;\text{x}\;100$$

Matthews correlation coefficient (MCC): considered to be the most robust parameter of any class prediction method. MCC equal to 1 is regarded as perfect prediction while 0 for completely random prediction.

(13) $$MCC=\frac{\left(TPxTN\right)-\left(FPxFN\right)}{\sqrt{\left(TP+FP\right)\left(TP+FN\right)\left(TN+FP\right)\left(TN+FN\right)}}$$

where TP = True Positives, TN = True Negatives, FP = False Positives, and FN = False Negatives.

In addition, we also plot Receiver Operating Characteristic (ROC) curves and calculate the Area Under Curve (AUC) for each of the classifiers.

## Results and discussion

At first, we will describe the homology-based prediction results and then, the SVM-based performance for both the phases of plastid-types prediction, including testing on independent datasets.

### (i). Homology-based PSI-BLAST

A biologist would always want to first check the similarity-based predcitions as is done usually in research labs. We performed PSI-BLAST of the 2844 positive set and 2844 negative set proteins against the UniProt/Swiss-Prot datatbase. Results in Table 5 show that, although the negative set proteins could be predicted with about 82% accuracy, the positive proteins are only correctly annotated with about 50% accuracy. About 1443 plastid preoteins are correctly predicted out of 2844. Thus a significant fraction of the positive set (~50%) could not be predicted using a homology-based approach. In phase-II, the performance of Psi-Blast was even worse. Only 167 chloropast proteins could be predicted correctly out of 542 in the query set with an accuracy of about 31% (Table 5). Other plastid-type results: chromoplast (9.61%), amyloplast (18.10%) and etioplast (1.82%) show that the similarity-based approach fails in characterizing various forms of plastids. Machine learning-based algorithms such as using the SVMs are thus a good alternative for prediction purposes. We describe here the SVM results in detail for both the steps separately.

**Table 5:** Overall performance of homology-based (PSI-BLAST) prediction for the identification of plastid vs. non-plastid proteins and the classification of diverse plastid-types.

	No. of sequences	H	C	P (%)	A (%)
**Phase-I:**					
**Plastids**	2844	2731	1443	52.84	50.74
**Non-plastids**	2844	2726	2337	85.73	82.17

**Phase-II:**					
**Chloroplast**	542	483	167	34.58	30.81
**Chromoplast**	177	172	17	9.88	9.61
**Etioplast**	220	204	4	1.96	1.82
**Amyloplast**	232	219	42	19.18	18.10

*at e-value = 0.001; H = Number of total hits; C = Number of correct or true hits; P = Percent of correct hits calculated as (C/H*100); A = Percent accuracy calculated as (C/total number of proteins in a particular class * 100).

### (ii). Phase-I: SVM-based identification of plastid proteins

**Figure 2:**
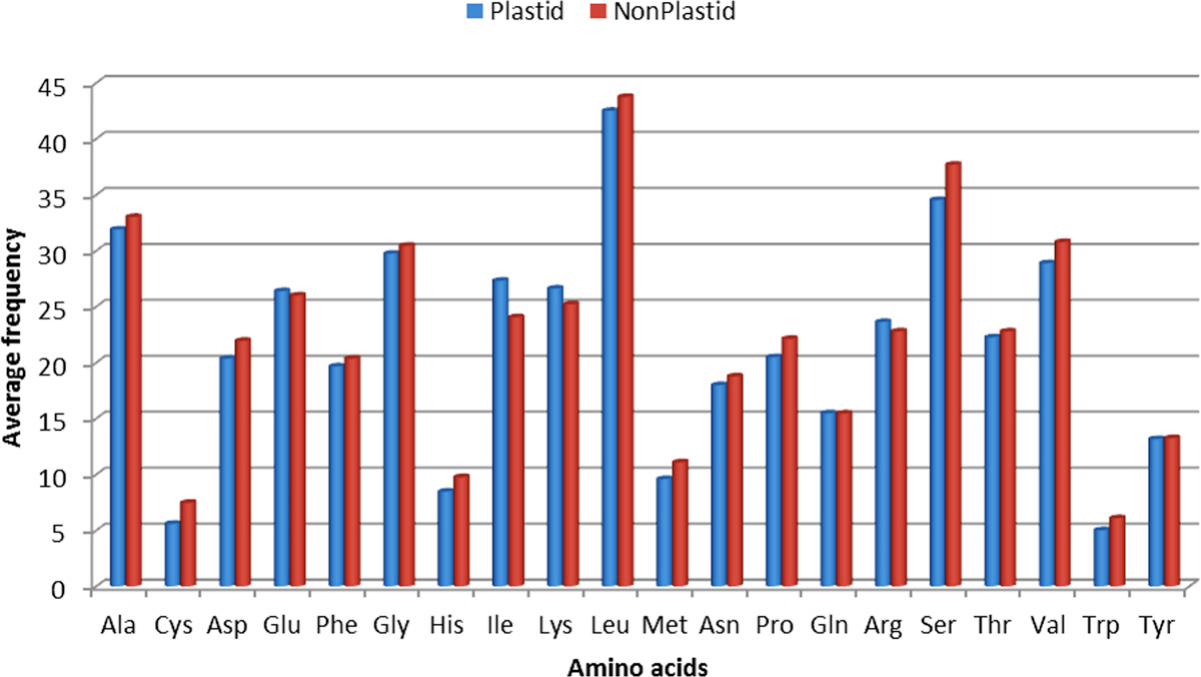
**A comaprative bar-graph of amino acid composition differences in plastid and non-plastid proteins**.

First, the amino acid frequencies of both plastid and non-plastid proteins were compared. Figure 2 shows a bar-graph comparing the amino acid frequencies of plastid and non-plastid proteins, concluding that there is a significant variation in both the compositions. The statistical significance of this variation was assessed with a p-value, estimated with a two-tailed Student's t-test (Additional file 1: Table S1). A summary of the observations as reported in Table S1 and Figure 2 indicate that the composition of 11 amino acids *viz*. Ala (A), Cys (C), Ile (I), Met (M), Pro (P), Val (V), Asp (D), His (H), Lys (K), Ser (S), and Trp (W) is significantly different in plastids and non-plastids. Secondly, to have more understanding in the variation of compositional features, we grouped the amino acids into seven classes based on the chemical and/or structural properties of their side chains *viz*. aliphatic, aromatic, acidic, basic, hydroxylic, Sulfur-containing, and amidic. We assessed the significance of difference by the t-test and listed in Table S2 (Additional file 1). The p-values at 0.05 level of significance shows that aromatic, hydroxylic and sulfur-containing amino acids vary significantly in plastids and non-plastids.

These two tests show that it is possible to develop various composition-based models for distinguishing plastid and non-plastid proteins. In a five-fold cross-validation approach, the simple amino acid composition-based model achieves a sensitivity of 85.37%, prediction accuracy of 85.51% with a MCC of 0.71 (Table 6). The precision rate is also more than 85%, which shows that plastid proteins could be predicted with a high positive prediction rate. Many researchers have reported the usefulness of amino acid composition for prediction purposes, e.g. in prediction of subcellular localization [49,50]; and how it carries a signal, almost entirely due to the surface residues that identifies the subcellular location [51]. Next, we developed a PseAAC classifier. The performance increased with a sensitivity of 89.45%, accuracy of 86.20% and a slight increase in the MCC (0.73) (Table 6). The PseAAC approach takes into consideration the composition, based on physicochemical properties and also includes the correlation factors associated with the protein chain, thus providing better and more dimensional information to the SVM.

**Table 6:** Overall performance of various feature classifiers in 5-fold cross-validation for the identification of plastid vs. non-plastid proteins (*phase-I*)

Feature type	Sensitivity (%)	Specificity (%)	Accuracy (%)	MCC	Precision (%)	RFP (%)	SVM kernel type
**AAC**	85.37	85.65	85.51	0.71	85.61	14.39	RBF (*γ *= 370, *C *= 3, *j *= 1)
**PseAA**	89.45	82.95	86.20	0.73	83.99	16.01	RBF (*γ *= 385, *C *= 2, *j *= 2)
**Dipep**	88.08	85.51	86.80	0.74	85.88	14.12	RBF (*γ *= 265, *C *= 6, *j *= 1)
**NCC**	84.14	89.66	86.90	0.74	89.06	10.94	RBF (*γ *= 20, *C *= 3, *j *= 2)
**Phys-Chem**	79.57	81.05	80.31	0.61	80.76	19.24	RBF (*γ *= 135, *C *= 2, *j *= 1)

*best values obtained at ≥ 0.0 threshold, individual performance of these classifiers can be seen in supplementary material; AAC = amino acid composition, PseAA = Pseudo amino acid composition, Dipep = Dipeptide composition, NCC = N_terminal_-Center-C_terminal _composition (*sequence divided into 3 parts*), Phys-Chem = Protein physicochemical properties, MCC = Matthews Correlation Coefficient, RFP = Rate of False Predictions, RBF = Radial Basis Function of SVM.

To include more diverse information, we further develop a dipeptide composition-based model. This classifier achieves the highest MCC (0.74) of all models with a slight increase in accuracy (86.80%) and a significant reduction in the rate of false prediction (14.12%). It has been reported in earlier studies that dipeptide composition performs better as compared to the simple amino acid composition [29,30,32], because it also provides the sequence order information along with the composition. Next, we compared the results of NCC and physicochemical property-based composition models. The physicochemical model, with an overall sensitivity of 79.57% and MCC of 0.61, did not perform well in predicting the plastid proteins comparatively. The NCC-based classifier achieves an accuracy of 86.90 % with a MCC of 0.74, which is at par with the DIPEP model, although the sensitivity was less in comparison. However, it achieves a higher specificity (89.66%) and precision (89.06%) value, with a lower RFP (10.94%) of all the models. Thus, for distinguishing plastid vs non-plastid proteins, both the DIPEP and NCC classifiers could be used efficiently, as both achieve the best MCC of 0.74 with higher accuracies (~87%). To check this further, we plot ROC curves for each of the models as discussed below. Please note: Table 6 is the overall performance of prediction modules at 0.0 threshold score of SVM. Individual performances of these classifiers at all values of threshold (-1.2 to 1.2) are available in the Supplementary Material (Additional file 1: Tables S3-S7).

**Figure 3:**
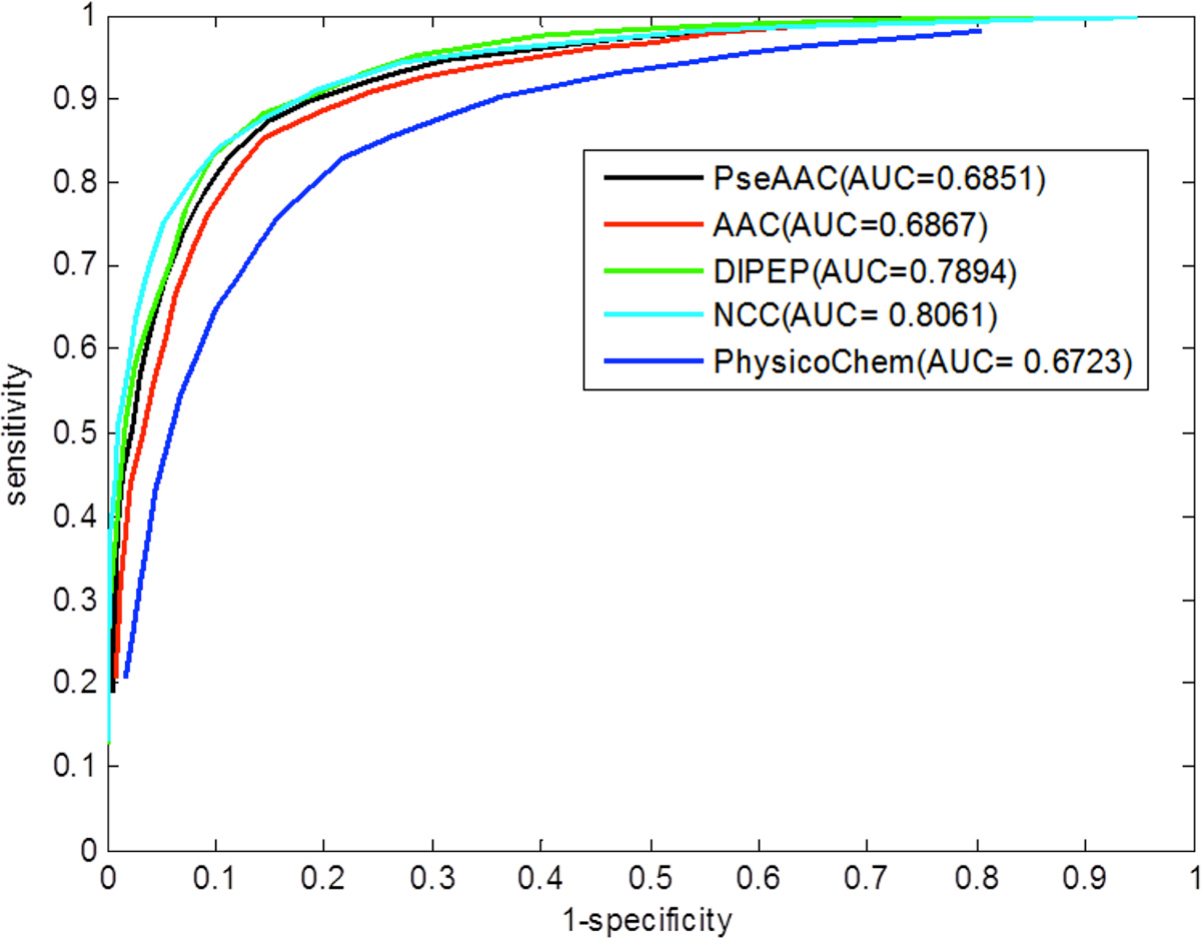
**ROC curve for all five classifiers (AAC, PseAAC, DIPEP, NCC, PhysicoChem) in phase-I prediction; plastid vs. non-plastid proteins identification**. AUC = Area Under Curve, AAC = amino acid composition, PseAAC = pseudo amino acid composition, DIPEP = dipeptide composition, NCC = N_terminal_-Center-C_terminal _composition, PhysicoChem = Protein physicochemical properties.

**ROC curves**: A plot of ROC curve is a statistical measure, which depicts the relationship between True Positive Rate (TPR) and the False Positive Rate (FPR, i.e. 1-specificity) for a binary classifier system as its discrimination threshold is varied. Figure 3 depicts the ROC curves for each of the five classifiers developed. It shows that the curves for DIPEP and NCC models are closer to the left side of the chart, primarily because they have very high specificity values at all the thresholds. This is a desirable characteristic of ROC curves. We also calculated the AUC values for each model (Figure 3), which shows that the AUC of 0.79 and 0.80 for the DIPEP and the NCC model, respectively are better than the others. The AUC specifies the probability that when we draw one positive and one negative example at random, the decision function assigns a higher value to the positive than to the negative example. So in phase-I prediction, we judged the DIPEP and NCC models as the best classifiers for predicting plastid vs. non-plastid proteins.

**Performance on independent set**: As mentioned in the methodology section, testing on independent datasets is considered to be another approach to judge the overall performance of a classifier, as they are not used in a machine learning process. Our independent set consists of 316 sequences each in positive as well as negative datasets. We run all five classifiers through these datasets separately. Table 7 shows that although the sensitivity values for AAC (69.30%), PseAAC (68.35%), NCC (65.82) and Physciochemical (68.35) model are higher than DIPEP (60.44%), they have lower specificity and precision values with a higher RFP. In machine learning, it is very important to have a balance between the sensitivity and specificity values to judge the overall performance of a classifier. The DIPEP model depicts the highest positive prediction rate of 89.25% with a very high specificity of 92.72%, which means that the RFP is the lowest (10.75%) of all the classifiers (Table 7). Accordingly, it would be wise to adjudge the DIPEP-based model as a better performing classifier. Individual performances of these five classifiers on independent test sets at all values of threshold (-1.2 to 1.2) are available in the Supplementary Material (Additional file 1: Table S13-S17).

**Table 7:** Overall performance of various feature classifiers on an '*independent test*' dataset for the identification of plastid vs. non-plastid proteins (*phase-I*)

Feature type	Sensitivity (%)	Specificity (%)	Accuracy (%)	MCC	Precision (%)	RFP (%)
**AAC**	69.30	87.03	78.16	0.57	84.23	15.77
**PseAA**	68.35	87.34	77.85	0.57	84.38	15.62
**Dipep**	60.44	92.72	76.58	0.56	89.25	10.75
**NCC**	65.82	87.97	76.90	0.55	84.55	15.45
**Phys-Chem**	68.35	84.49	76.42	0.54	81.51	18.49

*individual performance of these classifiers can be seen in supplementary material; AAC = amino acid composition (best values at ≥ 0.0 threshold), PseAA = Pseudo amino acid composition (best values at ≥ 0.1 threshold), Dipep = Dipeptide composition (best values at ≥ 0.2 threshold), NCC = N_terminal_-Center-C_terminal _composition (*sequence divided into 3 parts*), Phys-Chem = Protein physicochemical properties, MCC = Matthews Correlation Coefficient, RFP = Rate of False Predictions.

### (iii). Phase-II: SVM-based classification of plastid-type proteins

**Figure 4:**
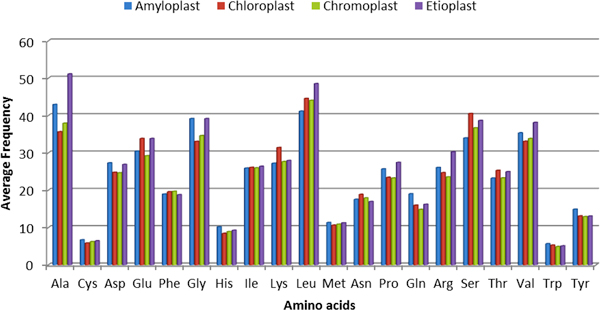
**A comparative bar-graph of amino acid composition differences among various plastid-types; amyloplast, chromoplast, chloroplast and etioplast proteins**.

In the current study, one of our major goals was to predict various plastid-types based on their function. So the proteins that are identified as plastids from phase-I would be further classified into one of its sub classes using the prediction models developed in phase-II. Similar to the phase-I, we first compared the amino acid compositions among various plastid types under study; chloroplast, chromoplast, etioplast and amyloplast (Figure 4). We assessed the significance of the amino acid compositions using Student's t-test and found that there exists a statistically significant variation in discriminating various plastid types. The p-values of the significance test are listed in Table S1 (Additional file 1). Secondly, as done in phase-I, we also compared the physicochemical property-based difference among the plastid types based on grouping the amino acids into seven classes (Table S2). Based on the t-test, we observed that the aliphatic, aromatic, acidic, basic and hydroxylic amino acids have significant variation in most of the plastid types. The above comparison shows that there exists a significant difference in compositions among various sub classes of plastids, which is used as a basis to develop various prediction models in this study.

The overall performance of the five multi-class models; AAC, PseAAC, DIPEP, NCC and Physicochemical-based is depicted in Table 8. The simple AAC model achieves a sensitivity of about 60% with an accuracy of 77.45% and precision 59%. The MCC is 0.40. Using PseAAC improved the results slightly, predicting plastid sub-classes with an overall accuracy of about 78% and MCC = 0.41. The NCC model show comparable results with an overall accuracy of 78.39%, sensitivity 60.97 % and MCC of 0.42. Comparatively, the physicochemical model achieves less accuracy with a sensitivity of 56.74% and MCC 0.36 only. However, we note that the DIPEP classifier again performs better as compared to the other features with an overall sensitivity of 62.26%, accuracy of 78.60% and a better MCC of 0.44. The precision rate is also high, about 63%. This shows that the DIPEP feature works well in both the phases of plastid prediction and can be used for annotation purposes. The performances of these five classifiers individually on each plastid-type category can be found in the Supplementary Material (Additional file 1: Table S8-S12).

**Table 8:** Overall performance of various feature classifiers in 5-fold cross-validation for the classification of diverse plastid-types^* ^(*phase-II*)

Feature type	Sensitivity (%)	Specificity (%)	Accuracy (%)	MCC	Precision (%)	ER (%)	SVM kernel type
**AAC**	60.03	76.05	77.45	0.40	59.00	22.55	RBF (*γ *= 246, *C *= 1, *j *= 2)
**PseAA**	60.72	77.13	78.01	0.41	59.55	21.99	RBF (*γ *= 225, *C *= 1, *j *= 2)
**Dipep**	62.26	75.85	78.60	0.44	62.62	21.40	RBF (*γ *= 210, *C *= 1, *j *= 2)
**NCC**	60.97	77.34	78.39	0.42	58.51	21.61	RBF (*γ *= 5, *C *= 2, *j *= 3)
**Phys-Chem**	56.70	78.01	76.56	0.36	54.15	23.44	RBF (*γ *= 37, *C *= 9, *j *= 1)

*classification of 4 plastid types: chloroplast, chromoplast, etioplast, amyloplast; individual performance of these classifiers on each class can be seen in supplementary material; AAC = amino acid composition, PseAA = Pseudo amino acid composition, Dipep = Dipeptide composition, NCC = N_terminal_-Center-C_terminal _composition (*sequence divided into 3 parts*), Phys-Chem = Protein physicochemical properties, MCC = Matthews Correlation Coefficient, ER = Error Rate, RBF = Radial Basis Function of SVM.

It is worth mentioning that prediction performance falls significantly in phase-II compared to the phase-I prediction process. This might be due to the fact that all of the sub-classes of plastids have common targeting signals (e.g. the transit peptides), as all still belong to one class 'plastids' and thus, it may be very difficult to distinguish their individual patterns by machine learning. However the overall amino acid composition varied significantly among them (Figure 4, and Additional file 2: Figure S1), which contributed towards respectable prediction accuracies as shown in Table 8. Combined, the results show that the plastid types could be categorized computationally with a statisfactory performance level. Although the models need more refinement, which we plan to do in the future, as, and when, more plastid-type training data is added to various repositories.

**Figure 5:**
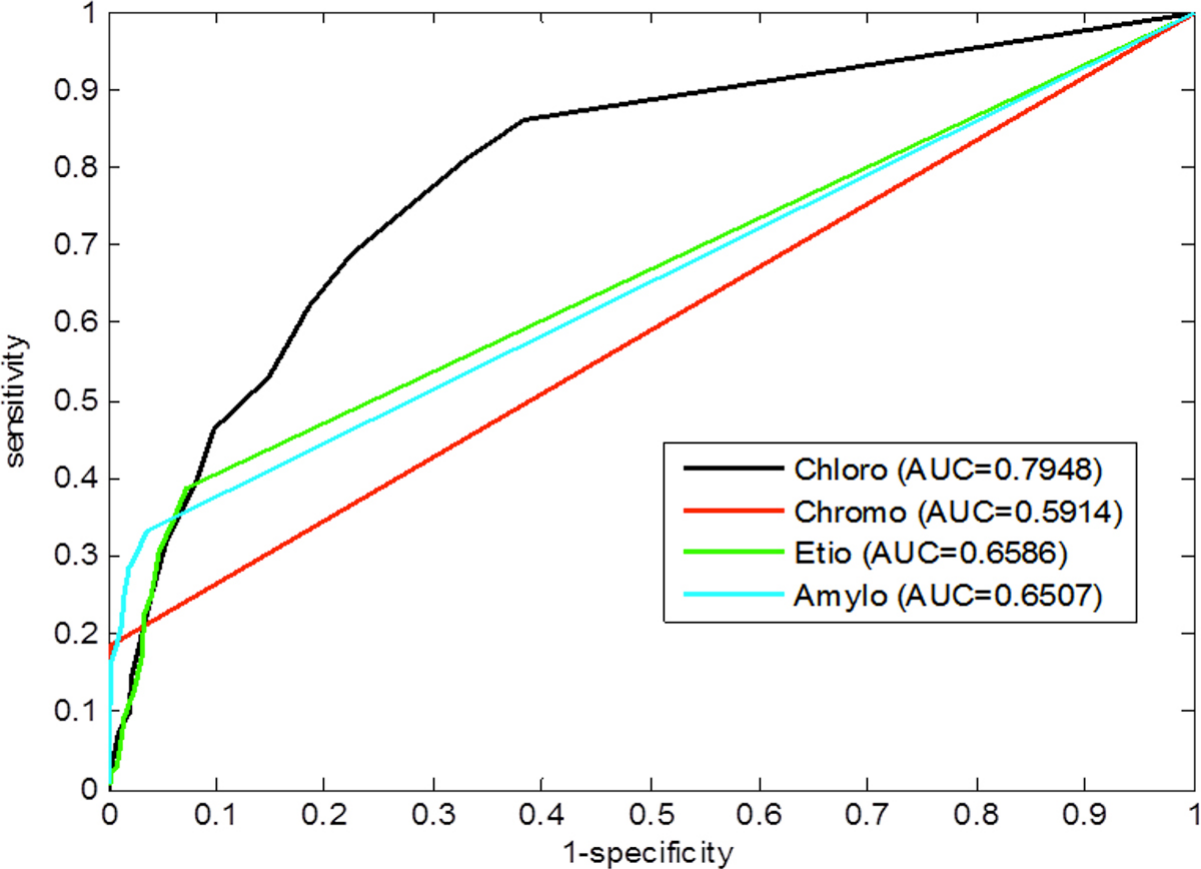
**ROC curves for the best classifier (Dipeptide composition-based) in phase-II prediction, i.e. classification of various plastid types (chloroplast, chromoplast, etioplast, amyloplast)**. Values in parentheses represent Area Under Curve (AUC).

**ROC curves**: Figure 5 shows the ROC curves for the four sub-classes of plastids. As the DIPEP-based model shows better performance in five-fold cross-validation, we use this classifier to draw ROC plots. As expected, the 'chloroplast' class shows a better ROC plot compared to other classes. A more precise way of evaluating the performance is to calculate the AUC. The closer the area to 0.5, the poorer the method, and the closer to 1.0, the better the method. The AUC for chloroplast (0.80) is the highest of all, which indicates that the "chloro" type plastids are more easily identifiable than other plastids. The other sub-classes *viz*. chromoplast (AUC = 0.59), etioplast (AUC = 0.66) and amyloplast (AUC = 0.65) achieve a satisfactory level of area under curve values.

**Performance on an independent dataset**: As in phase-I, we also tested the phase-II models on an independent dataset that contains 60 chloroplast sequences, 17 chromoplast, 24 etioplast and 23 amyloplast type proteins. The overall performance of each classifier is depicted in Table 9 and the individual performances on each subclass are available in the Supplementary Material (Additional file 1: Tables S18-S22). As with the 5-fold results, the DIPEP-based model outperformed the other classifiers and achieved an overall sensitivity of 61.29% with an accuracy of about 75%. The rate of positive class prediction, precision (~74%) was also high with the DIPEP feature (Table 9). The NCC-based classifier performed almost at par with the DIPEP model with the same sensitivity and MCC values, although with a lower precision value (60.42%).

**Table 9:** Overall performance of various feature classifiers on an '*independent test*' dataset for the classification of diverse plastid-types^* ^(*phase-II*)

Feature type	Sensitivity (%)	Specificity (%)	Accuracy (%)	MCC	Precision (%)	ER (%)
**AAC**	57.26	63.89	72.54	0.30	62.45	27.47
**PseAA**	57.26	63.88	72.48	0.31	65.25	27.52
**Dipep**	61.29	65.96	74.97	0.40	73.97	25.03
**NCC**	61.29	75.82	77.15	0.40	60.42	22.85
**Physico-Chem**	45.97	65.30	66.63	0.14	47.03	33.37

*classification of 4 plastid types: chloroplast, chromoplast, etioplast, amyloplast; individual performance of these classifiers on each class can be seen in supplementary material; AAC = amino acid composition, PseAA = Pseudo amino acid composition, Dipep = Dipeptide composition, NCC = N_terminal_-Center-C_terminal _composition (*sequence divided into 3 parts*), Physico-Chem = Protein physicochemical properties, MCC = Matthews Correlation Coefficient, ER = Error Rate.

Overall, the above results suggest that it is possible to categorize plastid proteins into various plastid-types using machine learning approaches with a moderate to high accuracy; the similarity-based module showed very low performance in this study. Although we achieved a significantly high prediction performance in phase-I to distinguish plastid vs. non-plastid proteins, the performances of the models developed in phase-II were not so outstanding. As this is a first attempt to develop prediction models for plastid types based on their function, we achieved a satisfactory level of accuracy. One possible reason for the lower success level is that very few training sequences are available in classes such as chromoplast, etioplast and amyloplast and are almost negligible in other subtypes. Although experimental proteomics approaches have generated a considerable amount of data, more training data is needed to develop highly accurate and efficient prediction models. A second possible reason is that there might be very small differences in sequences among plastid-types, making it very challenging for machine learning modules to distinguish among them. We were able to achieve about 79% prediction accuracy in phase-II with a MCC of 0.44 and precision of 63%, which shows that it is certainly possible to classify plastid-types through machine learning. With the increase in datasets and also by applying novel algorithmic approaches, we will refine these models in future and make available on the PLpred web server.

### (iv). Comparison with existing plastid localization predictors

Although there are no existing tools to predict plastid subtypes, there are some web tools available for predicting the plastid localized proteins from the primary sequence information. We compared the performance of our phase-I models in distinguishing the plastid vs. non-plastid proteins with two widely used tools TargetP [52] and WoLF PSORT [53] along with two other recently developed predictors; YLoc-HiRes [54] and iLoc-Plant [55]. The performance of these methods was compared using the same independent dataset containing 316 plastid and 316 non-plastid proteins (Table 10). As both DIPEP and NCC models from our phase-I achieved almost the same results, we used both these models for comparison; results are presented separately. Results in Table 10 show that our method achieves a higher prediction accuracy of about 77% with a MCC of 0.56 as compared to other tools. The MCC achieved by other four tools is between 0.32 and 0.44 with overall prediction accuracies around 66%, which is 11% lower than our method. Within the existing tools, TargetP and Wolf PSORT show better results than YLoc and iLoc-Plant in correctly identifying the plastid proteins by providing higher sensitivity. Although our method outperform all other methods compared in this study by achieving high values for all the evaluation parameters. Thus, PLpred can be used as an efficient tool for predicting plastid proteins.

**Table 10:** Overall performance comparison of our method with the existing web tools for predicting plastid proteins.

Tools	Sensitivity (%)	Specificity (%)	Accuracy (%)	MCC	Precision (%)	RFP (%)
**WoLF PSORT**	56.96	74.76	65.82	0.3223	69.50	30.50
**TargetP**	55.70	85.89	65.97	0.3998	88.44	11.56
**iLoc-PLant**	36.39	98.42	67.41	0.4438	95.83	4.17
**YLoc **(HighRes)	34.81	97.47	66.14	0.4142	93.22	6.78
**PLpred **(DIPEP)	60.44	92.72	76.58	0.56	89.25	10.75
**PLpred **(NCC)	65.82	87.97	76.90	0.55	84.55	15.45

Performance comparison done on an '*independent dataset*' that contains 316 plastid and 316 non-plastid proteins. MCC = Matthews Correlation Coefficient, RFP = Rate of False Predictions, DIPEP = Dipeptide composition-based classifier, NCC = N_terminal_-Center-C_terminal _composition-based classifier.

## Conclusion

Plastids, found in plants and algae, are the major site of manufacture and storage of important chemical compounds used by the cell. In plants, they are differentiated into various forms, depending upon which function they play in the cell such as the chloroplast, chromoplast, etioplast, amyloplast *etc*. Recent proteomics approaches have generated an adequate amount of protein data in each of these sub classes. However, large-scale plastid proteomics has become difficult and is nearing saturation due to several constraints as discussed. On the other hand, with the emphasis on genome sequencing and more and more data being generated rapidly, there is a need for accurate computational systems that could be used for genome-wide annotation of various plant genomes. To date, there is no prediction system that can be used to categorize plastid proteins into their various functional types. The current work is an attempt in that direction where we explore homology-based as well as machine learning approaches to classify plastid protein types.

The similarity-based approach showed very weak performance indicating the need and importance of machine learning algorithms. Our benchmark tests on diverse training and testing data showed that it is possible to develop prediction models to distinguishing various plastid-types just from their sequences. Our SVM-based method works in two phases; it first identifies a query protein as plastid or non-plastid with high accuracy and then, further classifies the identified sequences into one of the four plastid subclasses under study. Although we will be further refining the phase-II models with the increase in data availability, the current method should be applicable to the annotation of various available proteomes.

## List of abbreviations

SVM: Support Vector Machine; AAC: Amino acid composition; PseAAC: Pseudo amino acid composition; DIPEP: Dipeptide composition; MCC: Matthews correlation coefficient; RBF: Radial Basis Function; TP: True positive; TN: True negative; FP: False positive; FN: False negative.

## Competing interests

The authors declare that they have no competing financial interests.

## Authors' contributions

RK conceived the study, collected the data, developed algorithms, participated in its design and coordination and wrote the final manuscript. SSS and RV helped in model development, performed the calculations, figures and tables, and helped in drafting the original manuscript. TW helped in data analysis and setting up the prediction tool developed from this study. All authors read and approved the final manuscript.

## Supplementary Material

Additional file 1 Supplementary material; tables Click here for file

Additional file 2 Figure S1 Click here for file

## Declaration

Funding for the publication of this article has come from start-up funds account AA-1-51220, OSU.

This article has been published as part of *BMC Bioinformatics *Volume 14 Supplement 14, 2013: Proceedings of the Tenth Annual MCBIOS Conference. Discovery in a sea of data. The full contents of the supplement are available online at http://www.biomedcentral.com/bmcbioinformatics/supplements/14/S14.
