# Supplementary material for: Identification and characterization of plastid-type proteins from sequence-attributed features using machine learning
Source: BMC Bioinformatics. 2013 Oct 9;14(Suppl 14):S7. doi: 10.1186/1471-2105-14-S14-S7 (PMC3851450; doi:10.1186/1471-2105-14-S14-S7)
Supplement: Additional file 1 — Supplementary material; tables [file 1471-2105-14-S14-S7-S1.docx]

**Supplementary material**

Table S1: P-values from pair-wise Student’s t-test designating amino acid composition difference among the proteins in Phase-I (plastid vs. non-plastid) and Phase-II (various plastid types)

|  | **Phase-I** | **Phase-II** | | | | | |
| --- | --- | --- | --- | --- | --- | --- | --- |
| **Amino acid** | Plastid vs. Non-Plastid | Chloro-Chromo | Chloro-Etio | Chromo-Etio | Amylo-Chloro | Amylo-Chromo | Amylo-Etio |
| Ala (A) | 0.0594^*^ | 0.0000073^*^ | 0.9402648 | 0^*^ | 0.0059052^*^ | 0.0855214 | 0.0021148^*^ |
| Cys (C) | 0^*^ | 0.0000452^*^ | 0.0008455^*^ | 0.0883206 | 0.0008591^*^ | 0.846621 | 0.7560564 |
| Asp (D) | 3.87E-04^*^ | 0^*^ | 0.0000025^*^ | 0.0040725^*^ | 0.0000014^*^ | 0.6787778 | 0.895029 |
| Glu (E) | 0.5153 | 0^*^ | 0^*^ | 0.0012175^*^ | 0^*^ | 0.9469668 | 0.1470729 |
| Phe (F) | 0.1121 | 0.0000107^*^ | 0.0000004^*^ | 0.3560206 | 0.0000001^*^ | 0.1330526 | 0.9704008 |
| Gly (G) | 0.2127 | 0.0000043^*^ | 0.0083275^*^ | 0.0002883^*^ | 0.0044262^*^ | 0.2821914 | 0.7958082 |
| His (H) | 1.79E-09^*^ | 0.0000004^*^ | 0.0000154^*^ | 0.0487663^*^ | 0.00028^*^ | 0.4262735 | 0.4668231 |
| Ile (I) | 1.79E-09^*^ | 0^*^ | 0.0000005^*^ | 0.0660409 | 0^*^ | 0.1939523 | 0.7286479 |
| Lys (K) | 0.0167^*^ | 0^*^ | 0^*^ | 0.0719163 | 0^*^ | 0.1742472 | 0.629716 |
| Leu (L) | 0.1566 | 0^*^ | 0.0000267^*^ | 0.0037587^*^ | 0^*^ | 0.1912202 | 0.0163359^*^ |
| Met (M) | 1.48E-13^*^ | 0.0000001^*^ | 0.0000038^*^ | 0.0643579 | 0.0000009^*^ | 0.5936697 | 0.9761874 |
| Asn (N) | 0.078 | 0^*^ | 0^*^ | 0.3843666 | 0^*^ | 0.0926684 | 0.8068961 |
| Pro (P) | 2.52E-04^*^ | 0.0000007^*^ | 0.004325^*^ | 0.0000415^*^ | 0.0000605^*^ | 0.2421606 | 0.1872753 |
| Gln (Q) | 0.9643 | 0^*^ | 0.0000009^*^ | 0.0204481^*^ | 0.0073397^*^ | 0.0295772^*^ | 0.1586138 |
| Arg (R) | 0.076 | 0^*^ | 0.0039727^*^ | 0.0000063^*^ | 0.0000012^*^ | 0.4273429 | 0.019982^*^ |
| Ser (S) | 2.04E-05^*^ | 0^*^ | 0.0000001^*^ | 0.0254037^*^ | 0^*^ | 0.1263442 | 0.0619509 |
| Thr (T) | 0.2124 | 0^*^ | 0^*^ | 0.0084494^*^ | 0^*^ | 0.4773847 | 0.2457046 |
| Val (V) | 5.12E-04^*^ | 0.0000001^*^ | 0.0004365^*^ | 0.0002972^*^ | 0.0000009^*^ | 0.985473 | 0.1499854 |
| Trp (W) | 2.42E-14^*^ | 0.000006^*^ | 0.0000115^*^ | 0.08428 | 0.0003231^*^ | 0.7988453 | 0.549156 |
| Tyr (Y) | 0.7357 | 0.0000004^*^ | 0.000003^*^ | 0.0929585 | 0.0005224^*^ | 0.567143 | 0.1579615 |

^*^ significant at 0.05 level of significance; Chloro = Chloroplast, Chromo = Chromoplast, Etio = Etioplast, Amylo = Amyloplast.

Table S2: P-values from pair-wise Student’s t-test designating physicochemical property-based composition difference among the proteins in Phase-I (plastid vs. non-plastid) and Phase-II (various plastid types)

|  | **Phase-I** | **Phase-II** | | | | | |
| --- | --- | --- | --- | --- | --- | --- | --- |
| **Physicochemical property** | Plastid vs. Non-Plastid | Chloro-Chromo | Chloro-Etio | Chromo-Etio | Amylo-Chloro | Amylo-Chromo | Amylo-Etio |
| Aliphatic | 0.298340862 | 4.49E-08^*^ | 0.0012^*^ | 8.70E-05^*^ | 5.69E-07^*^ | 0.8305 | 0.0881 |
| Aromatic | 0.019306443^*^ | 1.01E-06^*^ | 4.02E-07^*^ | 0.1604 | 2.85E-06^*^ | 0.6308 | 0.5038 |
| Acidic | 0.232338892 | 4.17E-12^*^ | 3.83E-08^*^ | 0.0017^*^ | 1.21E-10^*^ | 0.8737 | 0.3699 |
| Basic | 0.401395176 | 2.63E-11^*^ | 2.24E-07^*^ | 0.0017^*^ | 4.42E-10^*^ | 0.9409 | 0.2743 |
| Hydroxylic | 0.001084629^*^ | 1.99E-10^*^ | 2.03E-08^*^ | 0.0145^*^ | 1.89E-13^*^ | 0.0022^*^ | 0.0012^*^ |
| Sulfur-containing | 4.30145E-26^*^ | 2.80E-07^*^ | 1.55E-05^*^ | 0.0603 | 4.46E-06^*^ | 0.655 | 0.91 |
| Amidic | 0.335211928 | 7.22E-10^*^ | 9.30E-09^*^ | 0.1093 | 1.01E-06^*^ | 0.3493 | 0.278 |

^*^ significant at 0.05 level of significance; Chloro = Chloroplast, Chromo = Chromoplast, Etio = Etioplast, Amylo = Amyloplast.

Table S3: Performance of amino acid composition-based classifier in 5-fold cross-validation for the identification of plastid vs. non-plastid proteins.

| **Threshold** | **Sensitivity**  **(%)** | **Specificity**  **(%)** | **Accuracy**  **(%)** | **MCC** | **Precision**  **(%)** | **RFP**  **(%)** |
| --- | --- | --- | --- | --- | --- | --- |
| -1.2 | 99.51 | 22.89 | 61.20 | 0.35 | 56.34 | 43.66 |
| -1.1 | 99.16 | 27.67 | 63.41 | 0.38 | 57.82 | 42.18 |
| -1.0 | 98.84 | 32.81 | 65.82 | 0.42 | 59.53 | 40.47 |
| -0.9 | 98.42 | 38.89 | 68.65 | 0.46 | 61.69 | 38.31 |
| -0.8 | 97.82 | 44.37 | 71.10 | 0.50 | 63.75 | 36.25 |
| -0.7 | 96.66 | 49.93 | 73.29 | 0.53 | 65.88 | 34.12 |
| -0.6 | 96.03 | 55.17 | 75.60 | 0.56 | 68.17 | 31.83 |
| -0.5 | 94.76 | 60.79 | 77.78 | 0.59 | 70.73 | 29.27 |
| -0.4 | 93.74 | 66.28 | 80.01 | 0.62 | 73.54 | 26.46 |
| -0.3 | 92.62 | 70.96 | 81.79 | 0.65 | 76.13 | 23.87 |
| -0.2 | 90.86 | 75.63 | 83.25 | 0.67 | 78.85 | 21.15 |
| -0.1 | 88.57 | 80.38 | 84.48 | 0.69 | 81.87 | 18.13 |
| **0.0** | **85.37** | **85.65** | **85.51** | **0.71** | **85.61** | **14.39** |
| 0.1 | 81.05 | 88.19 | 84.62 | 0.69 | 87.28 | 12.72 |
| 0.2 | 76.27 | 90.58 | 83.42 | 0.68 | 89.00 | 11.00 |
| 0.3 | 71.94 | 92.05 | 82.00 | 0.65 | 90.05 | 9.95 |
| 0.4 | 66.63 | 93.67 | 80.15 | 0.63 | 91.33 | 8.67 |
| 0.5 | 61.32 | 94.66 | 77.99 | 0.59 | 91.98 | 8.02 |
| 0.6 | 55.66 | 95.68 | 75.67 | 0.56 | 92.79 | 7.21 |
| 0.7 | 49.89 | 96.62 | 73.26 | 0.53 | 93.66 | 6.34 |
| 0.8 | 43.67 | 97.71 | 70.69 | 0.49 | 95.03 | 4.97 |
| 0.9 | 37.48 | 98.31 | 67.90 | 0.45 | 95.69 | 4.31 |
| 1.0 | 31.65 | 98.73 | 65.19 | 0.41 | 96.15 | 3.85 |
| 1.1 | 25.39 | 99.05 | 62.22 | 0.36 | 96.40 | 3.60 |
| 1.2 | 20.50 | 99.30 | 59.90 | 0.32 | 96.68 | 3.32 |

Best results at SVM Radial Basis Function (RBF) kernel (*γ* = 370, *C* = 3, *j* = 1); values in bold represent best threshold cutoff score; MCC = Matthews Correlation Coefficient, RFP = Rate of False Predictions.

Table S4: Performance of Pseudo amino acid composition-based classifier in 5-fold cross-validation for the identification of plastid vs. non-plastid proteins.

| **Threshold** | **Sensitivity**  **(%)** | **Specificity**  **(%)** | **Accuracy**  **(%)** | **MCC** | **Precision**  **(%)** | **RFP**  **(%)** |
| --- | --- | --- | --- | --- | --- | --- |
| -1.2 | 99.68 | 19.83 | 59.76 | 0.32 | 55.43 | 44.57 |
| -1.1 | 99.65 | 23.95 | 61.80 | 0.36 | 56.71 | 43.29 |
| -1.0 | 99.51 | 28.55 | 64.03 | 0.40 | 58.21 | 41.79 |
| -0.9 | 99.16 | 34.18 | 66.67 | 0.44 | 60.10 | 39.90 |
| -0.8 | 98.66 | 39.91 | 69.29 | 0.48 | 62.15 | 37.85 |
| -0.7 | 98.28 | 45.64 | 71.96 | 0.52 | 64.39 | 35.61 |
| -0.6 | 97.57 | 51.90 | 74.74 | 0.56 | 66.98 | 33.02 |
| -0.5 | 97.29 | 58.16 | 77.73 | 0.60 | 69.93 | 30.07 |
| -0.4 | 96.20 | 63.89 | 80.05 | 0.63 | 72.71 | 27.29 |
| -0.3 | 95.08 | 68.74 | 81.91 | 0.66 | 75.26 | 24.74 |
| -0.2 | 93.39 | 73.38 | 83.39 | 0.68 | 77.82 | 22.18 |
| -0.1 | 91.49 | 77.95 | 84.72 | 0.70 | 80.58 | 19.42 |
| **0.0** | **89.45** | **82.95** | **86.20** | **0.73** | **83.99** | **16.01** |
| 0.1 | 84.49 | 87.31 | 85.90 | 0.72 | 86.94 | 13.06 |
| 0.2 | 79.85 | 89.84 | 84.85 | 0.70 | 88.71 | 11.29 |
| 0.3 | 74.44 | 92.30 | 83.37 | 0.68 | 90.62 | 9.38 |
| 0.4 | 69.23 | 93.85 | 81.54 | 0.65 | 91.84 | 8.16 |
| 0.5 | 63.12 | 95.01 | 79.06 | 0.61 | 92.67 | 7.33 |
| 0.6 | 56.65 | 96.27 | 76.46 | 0.58 | 93.83 | 6.17 |
| 0.7 | 50.35 | 97.01 | 73.68 | 0.54 | 94.40 | 5.60 |
| 0.8 | 44.37 | 97.71 | 71.04 | 0.50 | 95.10 | 4.90 |
| 0.9 | 37.52 | 98.35 | 67.93 | 0.45 | 95.78 | 4.22 |
| 1.0 | 30.31 | 98.95 | 64.63 | 0.40 | 96.64 | 3.36 |
| 1.1 | 23.35 | 99.12 | 61.23 | 0.34 | 96.37 | 3.63 |
| 1.2 | 17.93 | 99.30 | 58.61 | 0.30 | 96.23 | 3.77 |

Best results at RBF kernel (*γ* = 385, *C* = 2, *j* = 2); values in bold represent best threshold cutoff score; MCC = Matthews Correlation Coefficient, RFP = Rate of False Predictions.

Table S5: Performance of Dipeptide composition-based classifier in 5-fold cross-validation for the identification of plastid vs. non-plastid proteins.

| **Threshold** | **Sensitivity**  **(%)** | **Specificity**  **(%)** | **Accuracy**  **(%)** | **MCC** | **Precision**  **(%)** | **RFP**  **(%)** |
| --- | --- | --- | --- | --- | --- | --- |
| -1.2 | 99.79 | 14.80 | 57.30 | 0.28 | 53.94 | 46.06 |
| -1.1 | 99.65 | 19.66 | 59.65 | 0.32 | 55.36 | 44.64 |
| -1.0 | 99.47 | 25.28 | 62.38 | 0.37 | 57.11 | 42.89 |
| -0.9 | 99.26 | 31.86 | 65.56 | 0.42 | 59.29 | 40.71 |
| -0.8 | 99.02 | 39.38 | 69.20 | 0.48 | 62.03 | 37.97 |
| -0.7 | 98.66 | 45.15 | 71.91 | 0.52 | 64.27 | 35.73 |
| -0.6 | 98.24 | 53.45 | 75.84 | 0.58 | 67.85 | 32.15 |
| -0.5 | 97.57 | 60.20 | 78.89 | 0.62 | 71.03 | 28.97 |
| -0.4 | 96.41 | 65.79 | 81.10 | 0.65 | 73.81 | 26.19 |
| -0.3 | 95.15 | 71.20 | 83.18 | 0.68 | 76.77 | 23.23 |
| -0.2 | 93.25 | 76.16 | 84.70 | 0.70 | 79.64 | 20.36 |
| -0.1 | 90.65 | 80.38 | 85.51 | 0.71 | 82.21 | 17.79 |
| **0.0** | **88.08** | **85.51** | **86.80** | **0.74** | **85.88** | **14.12** |
| 0.1 | 83.30 | 90.23 | 86.76 | 0.74 | 89.50 | 10.50 |
| 0.2 | 76.79 | 92.65 | 84.72 | 0.70 | 91.27 | 8.73 |
| 0.3 | 70.36 | 94.23 | 82.30 | 0.67 | 92.42 | 7.58 |
| 0.4 | 63.99 | 96.06 | 80.03 | 0.63 | 94.20 | 5.80 |
| 0.5 | 58.19 | 97.26 | 77.73 | 0.60 | 95.50 | 4.50 |
| 0.6 | 51.16 | 98.21 | 74.68 | 0.56 | 96.61 | 3.39 |
| 0.7 | 44.48 | 98.80 | 71.64 | 0.52 | 97.38 | 2.62 |
| 0.8 | 37.13 | 99.23 | 68.18 | 0.46 | 97.96 | 2.04 |
| 0.9 | 30.70 | 99.54 | 65.12 | 0.42 | 98.53 | 1.47 |
| 1.0 | 23.38 | 99.79 | 61.59 | 0.36 | 99.11 | 0.89 |
| 1.1 | 17.97 | 99.89 | 58.93 | 0.31 | 99.42 | 0.58 |
| 1.2 | 12.59 | 99.93 | 56.26 | 0.26 | 99.44 | 0.56 |

Best results at RBF kernel (*γ* = 265, *C* = 6, *j* = 1); values in bold represent best threshold cutoff score; MCC = Matthews Correlation Coefficient, RFP = Rate of False Predictions.

Table S6: Performance of NCC composition-based classifier in 5-fold cross-validation for the identification of plastid vs. non-plastid proteins.

| **Threshold** | **Sensitivity**  **(%)** | **Specificity**  **(%)** | **Accuracy**  **(%)** | **MCC** | **Precision**  **(%)** | **RFP**  **(%)** |
| --- | --- | --- | --- | --- | --- | --- |
| -1.2 | 99.79 | 5.13 | 52.46 | 0.15 | 51.26 | 48.74 |
| -1.1 | 99.68 | 8.86 | 54.27 | 0.20 | 52.24 | 47.76 |
| -1.0 | 99.47 | 13.29 | 56.38 | 0.25 | 53.43 | 46.57 |
| -0.9 | 99.23 | 19.20 | 59.21 | 0.31 | 55.12 | 44.88 |
| -0.8 | 99.05 | 27.22 | 63.13 | 0.38 | 57.64 | 42.36 |
| -0.7 | 98.59 | 35.83 | 67.21 | 0.44 | 60.57 | 39.43 |
| -0.6 | 97.96 | 45.68 | 71.82 | 0.51 | 64.33 | 35.67 |
| -0.5 | 97.01 | 55.27 | 76.14 | 0.58 | 68.44 | 31.56 |
| -0.4 | 95.78 | 64.59 | 80.19 | 0.64 | 73.01 | 26.99 |
| -0.3 | 94.16 | 72.86 | 83.51 | 0.69 | 77.62 | 22.38 |
| -0.2 | 91.03 | 80.52 | 85.78 | 0.72 | 82.37 | 17.63 |
| -0.1 | 87.62 | 85.58 | 86.60 | 0.73 | 85.87 | 14.13 |
| **0.0** | **84.14** | **89.66** | **86.90** | **0.74** | **89.06** | **10.94** |
| 0.1 | 79.96 | 92.37 | 86.16 | 0.73 | 91.29 | 8.71 |
| 0.2 | 74.93 | 94.90 | 84.92 | 0.71 | 93.63 | 6.37 |
| 0.3 | 69.55 | 96.13 | 82.84 | 0.68 | 94.73 | 5.27 |
| 0.4 | 63.47 | 97.40 | 80.43 | 0.65 | 96.06 | 3.94 |
| 0.5 | 57.10 | 98.10 | 77.60 | 0.61 | 96.78 | 3.22 |
| 0.6 | 50.84 | 98.87 | 74.86 | 0.57 | 97.83 | 2.17 |
| 0.7 | 44.41 | 99.19 | 71.80 | 0.52 | 98.21 | 1.79 |
| 0.8 | 38.22 | 99.54 | 68.88 | 0.48 | 98.82 | 1.18 |
| 0.9 | 30.59 | 99.72 | 65.15 | 0.42 | 99.09 | 0.91 |
| 1.0 | 23.84 | 99.79 | 61.81 | 0.36 | 99.12 | 0.88 |
| 1.1 | 18.11 | 99.82 | 58.97 | 0.31 | 99.04 | 0.96 |
| 1.2 | 13.05 | 99.96 | 56.50 | 0.26 | 99.73 | 0.27 |

Best results at SVM Radial Basis Function (RBF) kernel (*γ* = 20, *C* = 3, *j* = 2); values in bold represent best threshold cutoff score; NCC = N_terminal_-Center-C_terminal_ composition (*sequence divided into 3 parts*), MCC = Matthews Correlation Coefficient, RFP = Rate of False Predictions.

Table S7: Performance of Physiochemical property-based classifier in 5-fold cross-validation for the identification of plastid vs. non-plastid proteins.

| **Threshold** | **Sensitivity**  **(%)** | **Specificity**  **(%)** | **Accuracy**  **(%)** | **MCC** | **Precision**  **(%)** | **RFP**  **(%)** |
| --- | --- | --- | --- | --- | --- | --- |
| -1.2 | 98.14 | 19.51 | 58.83 | 0.29 | 54.94 | 45.06 |
| -1.1 | 97.47 | 24.30 | 60.88 | 0.32 | 56.28 | 43.72 |
| -1.0 | 96.87 | 29.57 | 63.22 | 0.36 | 57.90 | 42.10 |
| -0.9 | 96.31 | 35.09 | 65.70 | 0.40 | 59.74 | 40.26 |
| -0.8 | 95.46 | 40.96 | 68.21 | 0.43 | 61.79 | 38.21 |
| -0.7 | 94.44 | 46.45 | 70.45 | 0.47 | 63.82 | 36.18 |
| -0.6 | 93.14 | 52.67 | 72.91 | 0.50 | 66.31 | 33.69 |
| -0.5 | 91.60 | 58.16 | 74.88 | 0.53 | 68.64 | 31.36 |
| -0.4 | 90.15 | 63.78 | 76.97 | 0.56 | 71.34 | 28.66 |
| -0.3 | 87.97 | 68.88 | 78.43 | 0.58 | 73.87 | 26.13 |
| -0.2 | 85.55 | 73.73 | 79.64 | 0.60 | 76.51 | 23.49 |
| -0.1 | 82.88 | 78.20 | 80.54 | 0.61 | 79.17 | 20.83 |
| **0.0** | **79.57** | **81.05** | **80.31** | **0.61** | **80.76** | **19.24** |
| 0.1 | 76.02 | 84.14 | 80.08 | 0.60 | 82.74 | 17.26 |
| 0.2 | 72.26 | 86.04 | 79.15 | 0.59 | 83.81 | 16.19 |
| 0.3 | 68.64 | 87.97 | 78.31 | 0.58 | 85.09 | 14.91 |
| 0.4 | 64.77 | 89.87 | 77.32 | 0.56 | 86.48 | 13.52 |
| 0.5 | 59.60 | 91.60 | 75.60 | 0.54 | 87.64 | 12.36 |
| 0.6 | 54.54 | 93.11 | 73.82 | 0.52 | 88.78 | 11.22 |
| 0.7 | 48.66 | 94.37 | 71.52 | 0.48 | 89.64 | 10.36 |
| 0.8 | 43.14 | 95.53 | 69.34 | 0.45 | 90.62 | 9.38 |
| 0.9 | 37.24 | 96.13 | 66.68 | 0.41 | 90.59 | 9.41 |
| 1.0 | 31.93 | 96.94 | 64.43 | 0.38 | 91.26 | 8.74 |
| 1.1 | 25.98 | 97.64 | 61.81 | 0.34 | 91.69 | 8.31 |
| 1.2 | 20.75 | 98.17 | 59.46 | 0.30 | 91.90 | 8.10 |

Best results at SVM Radial Basis Function (RBF) kernel (*γ* = 135, *C* = 2, *j* = 1); values in bold represent best threshold cutoff score; MCC = Matthews Correlation Coefficient, RFP = Rate of False Predictions.

**Table S8.** Performance of simple amino acid (AA) composition-based classifier in 5-fold cross-validation for the classification of plastid-type proteins

| **Plastid type** | **Number of sequences** | **Sensitivity**  **(%)** | **Specificity**  **(%)** | **Accuracy**  **(%)** | **MCC** | **Precision**  **(%)** | **Error Rate (%)** |
| --- | --- | --- | --- | --- | --- | --- | --- |
| **Chloroplast** | 542 | 88.75 | 56.60 | 71.48 | 0.47 | 63.79 | 28.52 |
| **Chromoplast** | 177 | 20.90 | 97.79 | 86.17 | 0.31 | 62.71 | 13.83 |
| **Etioplast** | 220 | 43.18 | 90.12 | 81.30 | 0.35 | 50.27 | 18.70 |
| **Amyloplast** | 232 | 38.79 | 91.59 | 81.13 | 0.35 | 53.25 | 18.87 |
| **Overall** | **1171** | **60.03** | **76.05** | **77.45** | **0.40** | **59.00** | **22.55** |

Best results using RBF kernel (*γ* = 246, *C* = 1, *j* = 2).

**Table S9.** Performance of Pseudo amino acid composition-based classifier in 5-fold cross-validation for the classification of plastid-type proteins

| **Plastid type** | **Number of sequences** | **Sensitivity**  **(%)** | **Specificity**  **(%)** | **Accuracy**  **(%)** | **MCC** | **Precision**  **(%)** | **Error Rate (%)** |
| --- | --- | --- | --- | --- | --- | --- | --- |
| **Chloroplast** | 542 | 88.01 | 59.30 | 72.59 | 0.49 | 65.08 | 27.41 |
| **Chromoplast** | 177 | 23.16 | 97.49 | 86.25 | 0.32 | 62.12 | 13.75 |
| **Etioplast** | 220 | 46.36 | 89.91 | 81.73 | 0.38 | 51.52 | 18.28 |
| **Amyloplast** | 232 | 39.22 | 91.16 | 80.87 | 0.34 | 52.30 | 19.13 |
| **Overall** | **1171** | **60.72** | **77.13** | **78.01** | **0.41** | **59.55** | **21.99** |

Best results using RBF kernel (*γ* = 225, *C* = 1, *j* = 2).

**Table S10.** Performance of Dipeptide composition-based classifier in 5-fold cross-validation for the classification of plastid-type proteins

| **Plastid type** | **Number of sequences** | **Sensitivity**  **(%)** | **Specificity**  **(%)** | **Accuracy**  **(%)** | **MCC** | **Precision**  **(%)** | **Error Rate (%)** |
| --- | --- | --- | --- | --- | --- | --- | --- |
| **Chloroplast** | 542 | 93.73 | 54.53 | 72.67 | 0.52 | 63.98 | 27.33 |
| **Chromoplast** | 177 | 21.47 | 98.69 | 87.02 | 0.35 | 74.51 | 12.98 |
| **Etioplast** | 220 | 45.46 | 91.38 | 82.75 | 0.40 | 54.95 | 17.25 |
| **Amyloplast** | 232 | 35.78 | 93.50 | 82.07 | 0.36 | 57.64 | 17.93 |
| **Overall** | **1171** | **62.26** | **75.85** | **78.60** | **0.44** | **62.62** | **21.40** |

Best results using RBF kernel (*γ* = 210, *C* = 1, *j* = 2).

**Table S11.** Performance of NCC composition-based classifier in 5-fold cross-validation for the classification of plastid-type proteins

| **Plastid type** | **Number of sequences** | **Sensitivity**  **(%)** | **Specificity**  **(%)** | **Accuracy**  **(%)** | **MCC** | **Precision**  **(%)** | **Error Rate (%)** |
| --- | --- | --- | --- | --- | --- | --- | --- |
| **Chloroplast** | 542 | 89.67 | 59.46 | 73.44 | 0.51 | 65.59 | 26.56 |
| **Chromoplast** | 177 | 23.16 | 95.77 | 84.80 | 0.26 | 49.40 | 15.20 |
| **Etioplast** | 220 | 50.45 | 90.54 | 83.01 | 0.42 | 55.22 | 16.99 |
| **Amyloplast** | 232 | 32.76 | 92.55 | 80.70 | 0.31 | 52.05 | 19.30 |
| **Overall** | **1171** | **60.97** | **77.34** | **78.39** | **0.42** | **58.51** | **21.61** |

Best results using RBF kernel (*γ* = 5, *C* = 2, *j* = 3).

**Table S12.** Performance of Physiochemical property-based classifier in 5-fold cross-validation for the classification of plastid-type proteins

| **Plastid type** | **Number of sequences** | **Sensitivity**  **(%)** | **Specificity**  **(%)** | **Accuracy**  **(%)** | **MCC** | **Precision**  **(%)** | **Error Rate (%)** |
| --- | --- | --- | --- | --- | --- | --- | --- |
| **Chloroplast** | 542 | 82.29 | 64.07 | 72.50 | 0.47 | 66.37 | 27.50 |
| **Chromoplast** | 177 | 31.64 | 92.45 | 83.26 | 0.27 | 42.75 | 16.74 |
| **Etioplast** | 220 | 31.82 | 90.43 | 79.42 | 0.25 | 43.48 | 20.58 |
| **Amyloplast** | 232 | 39.66 | 87.75 | 78.22 | 0.29 | 44.44 | 21.78 |
| **Overall** | **1171** | **56.70** | **78.01** | **76.56** | **0.36** | **54.15** | **23.44** |

Best results using RBF kernel (*γ* = 37, *C* = 9, *j* = 1).

Table S13: Performance of amino acid composition-based classifier on an ‘*independent test*’ dataset for the identification of plastid vs. non-plastid proteins.

| **Threshold** | **Sensitivity**  **(%)** | **Specificity**  **(%)** | **Accuracy**  **(%)** | **MCC** | **Precision**  **(%)** | **RFP**  **(%)** |
| --- | --- | --- | --- | --- | --- | --- |
| -1.2 | 98.10 | 12.34 | 55.22 | 0.20 | 52.81 | 52.81 |
| -1.1 | 96.84 | 14.56 | 55.70 | 0.20 | 53.12 | 46.88 |
| -1.0 | 95.25 | 19.94 | 57.59 | 0.23 | 54.33 | 45.67 |
| -0.9 | 94.30 | 23.10 | 58.70 | 0.25 | 55.08 | 44.92 |
| -0.8 | 93.04 | 26.58 | 59.81 | 0.26 | 55.89 | 44.11 |
| -0.7 | 90.82 | 30.06 | 60.44 | 0.26 | 56.50 | 43.50 |
| -0.6 | 88.92 | 33.23 | 61.08 | 0.27 | 57.11 | 42.89 |
| -0.5 | 85.44 | 38.92 | 62.18 | 0.28 | 58.32 | 41.68 |
| -0.4 | 82.91 | 44.62 | 63.77 | 0.30 | 59.95 | 40.05 |
| -0.3 | 78.48 | 51.90 | 65.19 | 0.32 | 62.00 | 38.00 |
| -0.2 | 74.68 | 59.18 | 66.93 | 0.34 | 64.66 | 35.34 |
| -0.1 | 71.52 | 67.09 | 69.30 | 0.39 | 68.48 | 31.52 |
| **0.0** | **69.30** | **87.03** | **78.16** | **0.57** | **84.23** | **15.77** |
| 0.1 | 65.51 | 90.51 | 78.01 | 0.58 | 87.34 | 12.66 |
| 0.2 | 61.08 | 93.67 | 77.37 | 0.58 | 90.61 | 9.39 |
| 0.3 | 59.18 | 95.57 | 77.37 | 0.59 | 93.03 | 6.97 |
| 0.4 | 54.75 | 96.52 | 75.63 | 0.56 | 94.02 | 5.98 |
| 0.5 | 50.32 | 96.84 | 73.58 | 0.53 | 94.08 | 5.92 |
| 0.6 | 47.15 | 97.78 | 72.47 | 0.52 | 95.51 | 4.49 |
| 0.7 | 43.35 | 98.42 | 70.89 | 0.50 | 96.48 | 3.52 |
| 0.8 | 38.29 | 99.05 | 68.67 | 0.47 | 97.58 | 2.42 |
| 0.9 | 33.86 | 99.68 | 66.77 | 0.45 | 99.07 | 0.93 |
| 1.0 | 30.38 | 100.00 | 65.19 | 0.42 | 100.00 | 0.00 |
| 1.1 | 24.37 | 100.00 | 62.18 | 0.37 | 100.00 | 0.00 |
| 1.2 | 17.41 | 100.00 | 58.70 | 0.31 | 100.00 | 0.00 |

Values in bold represent best threshold cutoff score; MCC = Matthews Correlation Coefficient, RFP = Rate of False Predictions.

Table S14: Performance of Pseudo amino acid composition-based classifier on an ‘*independent test*’ dataset for the identification of plastid vs. non-plastid proteins.

| **Threshold** | **Sensitivity**  **(%)** | **Specificity**  **(%)** | **Accuracy**  **(%)** | **MCC** | **Precision**  **(%)** | **RFP**  **(%)** |
| --- | --- | --- | --- | --- | --- | --- |
| -1.2 | 98.42 | 6.65 | 52.53 | 0.13 | 51.32 | 48.68 |
| -1.1 | 97.78 | 9.18 | 53.48 | 0.15 | 51.85 | 48.15 |
| -1.0 | 97.47 | 10.76 | 54.11 | 0.17 | 52.20 | 47.80 |
| -0.9 | 95.89 | 12.97 | 54.43 | 0.16 | 52.42 | 47.58 |
| -0.8 | 94.62 | 19.62 | 57.12 | 0.22 | 54.07 | 45.93 |
| -0.7 | 92.72 | 22.47 | 57.59 | 0.21 | 54.46 | 45.54 |
| -0.6 | 90.51 | 25.63 | 58.07 | 0.21 | 54.89 | 45.11 |
| -0.5 | 88.61 | 29.75 | 59.18 | 0.23 | 55.78 | 44.22 |
| -0.4 | 85.44 | 37.03 | 61.23 | 0.26 | 57.57 | 42.43 |
| -0.3 | 82.28 | 44.94 | 63.61 | 0.29 | 59.91 | 40.09 |
| -0.2 | 80.06 | 50.95 | 65.51 | 0.32 | 62.01 | 37.99 |
| -0.1 | 75.32 | 58.54 | 66.93 | 0.34 | 64.50 | 35.50 |
| **0.0** | **72.47** | **70.57** | **71.52** | **0.43** | **71.12** | **28.88** |
| 0.1 | 68.35 | 87.34 | 77.85 | 0.57 | 84.38 | 15.62 |
| 0.2 | 65.82 | 89.24 | 77.53 | 0.57 | 85.95 | 14.05 |
| 0.3 | 62.34 | 91.77 | 77.06 | 0.57 | 88.34 | 11.66 |
| 0.4 | 57.28 | 94.30 | 75.79 | 0.56 | 90.95 | 9.05 |
| 0.5 | 53.16 | 96.52 | 74.84 | 0.55 | 93.85 | 6.15 |
| 0.6 | 49.37 | 97.78 | 73.58 | 0.54 | 95.71 | 4.29 |
| 0.7 | 44.62 | 98.10 | 71.36 | 0.51 | 95.92 | 4.08 |
| 0.8 | 38.92 | 98.73 | 68.83 | 0.47 | 96.85 | 3.15 |
| 0.9 | 35.44 | 99.37 | 67.41 | 0.45 | 98.25 | 1.75 |
| 1.0 | 30.06 | 100.00 | 65.03 | 0.42 | 100.00 | 0.00 |
| 1.1 | 25.00 | 100.00 | 62.50 | 0.38 | 100.00 | 0.00 |
| 1.2 | 18.99 | 100.00 | 59.49 | 0.32 | 100.00 | 0.00 |

Values in bold represent best threshold cutoff score; MCC = Matthews Correlation Coefficient, RFP = Rate of False Predictions.

Table S15: Performance of Dipeptide composition-based classifier on an ‘*independent test*’ dataset for the identification of plastid vs. non-plastid proteins.

| **Threshold** | **Sensitivity**  **(%)** | **Specificity**  **(%)** | **Accuracy**  **(%)** | **MCC** | **Precision**  **(%)** | **RFP**  **(%)** |
| --- | --- | --- | --- | --- | --- | --- |
| -1.2 | 98.10 | 7.59 | 52.85 | 0.13 | 51.50 | 48.50 |
| -1.1 | 97.15 | 10.44 | 53.80 | 0.15 | 52.03 | 47.97 |
| -1.0 | 95.57 | 13.61 | 54.59 | 0.16 | 52.52 | 47.48 |
| -0.9 | 94.62 | 16.14 | 55.38 | 0.17 | 53.01 | 53.01 |
| -0.8 | 93.99 | 20.25 | 57.12 | 0.21 | 54.10 | 45.90 |
| -0.7 | 91.46 | 27.53 | 59.49 | 0.25 | 55.79 | 44.21 |
| -0.6 | 89.24 | 34.18 | 61.71 | 0.28 | 57.55 | 42.45 |
| -0.5 | 86.08 | 40.51 | 63.29 | 0.30 | 59.13 | 40.87 |
| -0.4 | 82.91 | 45.57 | 64.24 | 0.31 | 60.37 | 39.63 |
| -0.3 | 80.70 | 53.16 | 66.93 | 0.35 | 63.28 | 36.72 |
| -0.2 | 75.00 | 59.18 | 67.09 | 0.35 | 64.75 | 35.25 |
| -0.1 | 70.89 | 67.72 | 69.30 | 0.39 | 68.71 | 31.29 |
| **0.0** | **67.09** | **77.85** | **72.47** | **0.45** | **75.18** | **24.82** |
| 0.1 | 63.92 | 88.92 | 76.42 | 0.55 | 85.23 | 14.77 |
| 0.2 | 60.44 | 92.72 | 76.58 | 0.56 | 89.25 | 10.75 |
| 0.3 | 55.06 | 93.99 | 74.53 | 0.53 | 90.16 | 9.84 |
| 0.4 | 51.58 | 96.52 | 74.05 | 0.54 | 93.68 | 6.32 |
| 0.5 | 46.84 | 98.73 | 72.78 | 0.53 | 97.37 | 2.63 |
| 0.6 | 37.97 | 98.73 | 68.35 | 0.46 | 96.77 | 3.23 |
| 0.7 | 32.28 | 99.37 | 65.82 | 0.43 | 98.08 | 98.08 |
| 0.8 | 26.58 | 99.68 | 63.13 | 0.38 | 98.82 | 98.82 |
| 0.9 | 20.89 | 100.00 | 60.44 | 0.34 | 100.00 | 0.00 |
| 1.0 | 16.46 | 100.00 | 58.23 | 0.30 | 100.00 | 0.00 |
| 1.1 | 12.34 | 100.00 | 56.17 | 0.26 | 100.00 | 0.00 |
| 1.2 | 10.13 | 100.00 | 55.06 | 0.23 | 100.00 | 0.00 |

Values in bold represent best threshold cutoff score; MCC = Matthews Correlation Coefficient, RFP = Rate of False Predictions.

Table S16. Performance of NCC composition-based classifier on an ‘*independent test*’ dataset for the identification of plastid vs. non-plastid proteins.

| **Threshold** | **Sensitivity**  **(%)** | **Specificity**  **(%)** | **Accuracy**  **(%)** | **MCC** | **Precision**  **(%)** | **RFP**  **(%)** |
| --- | --- | --- | --- | --- | --- | --- |
| -1.2 | 99.37 | 3.80 | 51.58 | 0.11 | 50.81 | 49.19 |
| -1.1 | 99.37 | 6.65 | 53.01 | 0.16 | 51.56 | 48.44 |
| -1.0 | 98.42 | 10.13 | 54.27 | 0.18 | 52.27 | 47.73 |
| -0.9 | 97.78 | 15.19 | 56.49 | 0.23 | 53.55 | 46.45 |
| -0.8 | 97.15 | 23.73 | 60.44 | 0.31 | 56.02 | 43.98 |
| -0.7 | 95.25 | 30.06 | 62.66 | 0.33 | 57.66 | 42.34 |
| -0.6 | 92.41 | 37.97 | 65.19 | 0.36 | 59.84 | 40.16 |
| -0.5 | 88.61 | 46.52 | 67.56 | 0.39 | 62.36 | 37.64 |
| -0.4 | 84.49 | 58.23 | 71.36 | 0.44 | 66.92 | 33.08 |
| -0.3 | 78.48 | 68.99 | 73.73 | 0.48 | 71.68 | 28.32 |
| -0.2 | 73.73 | 81.01 | 77.37 | 0.55 | 79.52 | 20.48 |
| -0.1 | 68.99 | 84.49 | 76.74 | 0.54 | 81.65 | 18.35 |
| **0.0** | **65.82** | **87.97** | **76.90** | **0.55** | **84.55** | **15.45** |
| 0.1 | 58.86 | 91.77 | 75.32 | 0.54 | 87.74 | 12.26 |
| 0.2 | 52.53 | 93.35 | 72.94 | 0.50 | 88.77 | 11.23 |
| 0.3 | 49.37 | 95.89 | 72.63 | 0.51 | 92.31 | 7.69 |
| 0.4 | 44.94 | 97.15 | 71.04 | 0.49 | 94.04 | 5.96 |
| 0.5 | 41.14 | 97.78 | 69.46 | 0.47 | 94.89 | 5.11 |
| 0.6 | 36.08 | 98.42 | 67.25 | 0.44 | 95.80 | 4.20 |
| 0.7 | 31.33 | 99.37 | 65.35 | 0.42 | 98.02 | 1.98 |
| 0.8 | 25.95 | 99.68 | 62.82 | 0.38 | 98.80 | 1.20 |
| 0.9 | 19.62 | 99.68 | 59.65 | 0.32 | 98.41 | 1.59 |
| 1.0 | 17.09 | 100.00 | 58.54 | 0.31 | 100.00 | 0.00 |
| 1.1 | 12.66 | 100.00 | 56.33 | 0.26 | 100.00 | 0.00 |
| 1.2 | 10.44 | 100.00 | 55.22 | 0.23 | 100.00 | 0.00 |

Values in bold represent best threshold cutoff score; NCC = N_terminal_-Center-C_terminal_ composition (*sequence divided into 3 parts*), MCC = Matthews Correlation Coefficient, RFP = Rate of False Predictions.

Table S17: Performance of Physiochemical property-based classifier on an ‘*independent test*’ dataset for the identification of plastid vs. non-plastid proteins.

| **Threshold** | **Sensitivity**  **(%)** | **Specificity**  **(%)** | **Accuracy**  **(%)** | **MCC** | **Precision**  **(%)** | **RFP**  **(%)** |
| --- | --- | --- | --- | --- | --- | --- |
| -1.2 | 97.47 | 12.03 | 54.75 | 0.18 | 52.56 | 47.44 |
| -1.1 | 97.15 | 18.99 | 58.07 | 0.26 | 54.53 | 45.47 |
| -1.0 | 95.89 | 22.78 | 59.34 | 0.27 | 55.39 | 44.61 |
| -0.9 | 93.67 | 25.95 | 59.81 | 0.27 | 55.85 | 44.15 |
| -0.8 | 89.87 | 29.75 | 59.81 | 0.25 | 56.13 | 43.87 |
| -0.7 | 87.66 | 35.44 | 61.55 | 0.27 | 57.59 | 42.41 |
| -0.6 | 84.81 | 42.41 | 63.61 | 0.30 | 59.56 | 40.44 |
| -0.5 | 82.59 | 47.78 | 65.19 | 0.32 | 61.27 | 38.73 |
| -0.4 | 80.06 | 52.85 | 66.46 | 0.34 | 62.94 | 37.06 |
| -0.3 | 78.16 | 60.44 | 69.30 | 0.39 | 66.40 | 33.60 |
| -0.2 | 75.32 | 65.19 | 70.25 | 0.41 | 68.39 | 31.61 |
| -0.1 | 70.89 | 81.01 | 75.95 | 0.52 | 78.87 | 21.13 |
| **0.0** | **68.35** | **84.49** | **76.42** | **0.54** | **81.51** | **18.49** |
| 0.1 | 65.51 | 86.39 | 75.95 | 0.53 | 82.80 | 17.20 |
| 0.2 | 62.66 | 88.92 | 75.79 | 0.53 | 84.98 | 15.02 |
| 0.3 | 58.23 | 90.82 | 74.53 | 0.52 | 86.38 | 13.62 |
| 0.4 | 53.80 | 92.41 | 73.10 | 0.50 | 87.63 | 12.37 |
| 0.5 | 48.42 | 94.62 | 71.52 | 0.49 | 90.00 | 10.00 |
| 0.6 | 44.94 | 95.25 | 70.09 | 0.47 | 90.45 | 9.55 |
| 0.7 | 40.82 | 95.89 | 68.35 | 0.44 | 90.85 | 9.15 |
| 0.8 | 36.39 | 96.84 | 66.61 | 0.42 | 92.00 | 8.00 |
| 0.9 | 32.91 | 98.42 | 65.66 | 0.41 | 95.41 | 4.59 |
| 1.0 | 30.06 | 98.42 | 64.24 | 0.39 | 95.00 | 5.00 |
| 1.1 | 26.27 | 98.73 | 62.50 | 0.36 | 95.40 | 4.60 |
| 1.2 | 20.89 | 99.05 | 59.97 | 0.32 | 95.65 | 4.35 |

Values in bold represent best threshold cutoff score; MCC = Matthews Correlation Coefficient, RFP = Rate of False Predictions.

**Table S18.** Performance of simple amino acid (AA) composition-based classifier on an ‘*independent test*’ dataset for the classification of plastid-type proteins

| **Plastid type** | **Number of sequences** | **Sensitivity**  **(%)** | **Specificity**  **(%)** | **Accuracy**  **(%)** | **MCC** | **Precision**  **(%)** | **Error Rate (%)** |
| --- | --- | --- | --- | --- | --- | --- | --- |
| **Chloroplast** | 60 | 93.33 | 28.125 | 59.67 | 0.28 | 54.90 | 40.32 |
| **Chromoplast** | 17 | 17.64 | 100 | 88.70 | 0.39 | 100 | 11.29 |
| **Etioplast** | 24 | 41.66 | 95 | 84.67 | 0.44 | 66.66 | 15.32 |
| **Amyloplast** | 23 | 8.69 | 98.01 | 81.45 | 0.14 | 50 | 18.54 |
| **Overall** | **124** | **57.25** | **63.88** | **72.53** | **0.30** | **62.45** | **27.46** |

**Table S19.** Performance of Pseudo amino acid composition-based classifier on an ‘*independent test*’ dataset for the classification of plastid-type proteins

| **Plastid type** | **Number of sequences** | **Sensitivity**  **(%)** | **Specificity**  **(%)** | **Accuracy**  **(%)** | **MCC** | **Precision**  **(%)** | **Error Rate (%)** |
| --- | --- | --- | --- | --- | --- | --- | --- |
| **Chloroplast** | 60 | 93.33 | 28.125 | 59.67 | 0.28 | 54.90 | 40.32 |
| **Chromoplast** | 17 | 23.52 | 100 | 89.51 | 0.45 | 100 | 10.48 |
| **Etioplast** | 24 | 33.33 | 94 | 82.25 | 0.34 | 57.14 | 17.74 |
| **Amyloplast** | 23 | 13.04 | 99.00 | 83.06 | 0.26 | 75 | 16.93 |
| **Overall** | **124** | **57.25** | **63.87** | **72.47** | **0.31** | **65.24** | **27.52** |

**Table S20.** Performance of Dipeptide composition-based classifier on an ‘*independent test*’ dataset for the classification of plastid-type proteins

| **Plastid type** | **Number of sequences** | **Sensitivity**  **(%)** | **Specificity**  **(%)** | **Accuracy**  **(%)** | **MCC** | **Precision**  **(%)** | **Error Rate (%)** |
| --- | --- | --- | --- | --- | --- | --- | --- |
| **Chloroplast** | 60 | 96.66 | 31.25 | 62.90 | 0.36 | 56.86 | 37.09 |
| **Chromoplast** | 17 | 23.52 | 100 | 89.51 | 0.45 | 100 | 10.48 |
| **Etioplast** | 24 | 45.83 | 96 | 86.29 | 0.50 | 73.33 | 13.70 |
| **Amyloplast** | 23 | 13.04 | 100 | 83.87 | 0.32 | 100 | 16.12 |
| **Overall** | **124** | **61.29** | **65.95** | **74.96** | **0.39** | **73.96** | **25.03** |

**Table S21.** Performance of NCC composition-based classifier on an ‘*independent test*’ dataset for the classification of plastid-type proteins

| **Plastid type** | **Number of sequences** | **Sensitivity**  **(%)** | **Specificity**  **(%)** | **Accuracy**  **(%)** | **MCC** | **Precision**  **(%)** | **Error Rate (%)** |
| --- | --- | --- | --- | --- | --- | --- | --- |
| **Chloroplast** | 60 | 83.33 | 57.81 | 70.16 | 0.42 | 64.94 | 29.84 |
| **Chromoplast** | 17 | 35.29 | 97.20 | 88.71 | 0.43 | 66.67 | 11.29 |
| **Etioplast** | 24 | 50.00 | 92.00 | 83.87 | 0.45 | 60.00 | 16.13 |
| **Amyloplast** | 23 | 34.78 | 90.10 | 79.84 | 0.27 | 44.44 | 20.16 |
| **Overall** | **124** | **61.29** | **75.82** | **77.15** | **0.40** | **60.42** | **22.85** |

NCC = N_terminal_-Center-C_terminal_ composition (*sequence divided into 3 parts*), MCC = Matthews Correlation Coefficient.

**Table S22.** Performance of Physiochemical property-based classifier on an ‘*independent test*’ dataset for the classification of plastid-type proteins

| **Plastid type** | **Number of sequences** | **Sensitivity**  **(%)** | **Specificity**  **(%)** | **Accuracy**  **(%)** | **MCC** | **Precision**  **(%)** | **Error Rate (%)** |
| --- | --- | --- | --- | --- | --- | --- | --- |
| **Chloroplast** | 60 | 70.00 | 39.06 | 54.03 | 0.10 | 51.85 | 45.97 |
| **Chromoplast** | 17 | 17.65 | 99.07 | 87.90 | 0.33 | 75.00 | 12.10 |
| **Etioplast** | 24 | 29.17 | 87.00 | 75.81 | 0.17 | 35.00 | 24.19 |
| **Amyloplast** | 23 | 21.74 | 86.14 | 74.19 | 0.09 | 26.32 | 25.81 |
| **Overall** | **124** | **45.97** | **65.30** | **66.63** | **0.14** | **47.03** | **33.37** |
