# Supplementary material for: Identification and characterization of plastid-type proteins from sequence-attributed features using machine learning
Source: BMC Bioinformatics. 2013 Oct 9;14(Suppl 14):S7. doi: 10.1186/1471-2105-14-S14-S7 (PMC3851450; doi:10.1186/1471-2105-14-S14-S7)
Supplement: Additional file 2 — Figure S1 [file 1471-2105-14-S14-S7-S2.docx]

Figure S1: A comaprative bar-graph of protein physicochemical properties showing significant difference among various plastid-types; amyloplast, chromoplast, chloroplast and etioplast proteins. Amino acids are grouped into 7 classes based on the chemical and/or structural properties of their side chains.
